# A metagenomic analysis of the bacterial microbiome of limestone, and the role of associated biofilms in the biodeterioration of heritage stone surfaces

**DOI:** 10.1038/s41598-022-08851-4

**Published:** 2022-03-22

**Authors:** Philip J. A. Skipper, Lynda K. Skipper, Ronald A. Dixon

**Affiliations:** 1 School of History and Heritage, University of Lincoln, Lincoln, UK; 2 School of Life Sciences, University of Lincoln, Lincoln, UK

**Keywords:** Biofilms, Next-generation sequencing, Microbiome

## Abstract

There is growing concern surrounding the aesthetic and physical effects of microbial biofilms on heritage buildings and monuments. Carboniferous stones, such as limestone and marble, are soluble in weak acid solutions and therefore particularly vulnerable to biocorrosion. This paper aims to determine the differences and commonalities between the microbiome of physically damaged and undamaged Lincolnshire limestone, an area of research which has not been previously studied. A lack of information about the core microbiome has resulted in conflicting claims in the literature regarding the biodeteriorative potential of many microorganisms. To address this, we used metagenomics alongside traditional microbiological techniques to produce an in-depth analysis of differences between the bacterial microbiomes found on deteriorated and undamaged external limestone surfaces. We demonstrate there is a core microbiome on Lincolnshire limestone present on both damaged and undamaged surfaces. In addition to the core microbiome, significant differences were found between species isolated from undamaged compared to damaged surfaces. Isolated species were characterised for biofilm formation and biodeteriorative processes, resulting in the association of species with biodeterioration that had not been previously described. Additionally, we have identified a previously undescribed method of biofilm-associated biomechanical damage. This research adds significant new understanding to the field, aiding decision making in conservation of stone surfaces.

## Introduction

The deterioration seen on stone surfaces results from chemical, physical and biological processes^1^. Biological processes of decay range from the impact of plants to bird droppings, but the important role of colonisation of the stone surface by microorganisms, such as bacteria, fungi and algae, through the formation of biofilms is probably underestimated. A biofilm is a protective matrix, a community of microorganisms which excrete materials, usually proteins and sugar polymers, to produce an extracellular polysaccharide matrix (EPS)^2^. Many bacterial species will spend at least part of their life cycle in biofilm mode, and the EPS protects the individual microorganisms from desiccation and physical damage while providing a greater surface area for them to occupy. Recent studies have identified biofilms containing bacteria, algae and fungi from many sources of historically relevant stonework including Mediterranean statuary^3–5^.

In England alone there are currently over 500,000 listed buildings. These are buildings which have been identified as nationally or internationally important, and worthy of preservation^6^. A search of the listed buildings database showed that as of November 2020, 36,616 of these listings relate to heritage sites composed partially or entirely of limestone and is an important resource for future generations. It is important that the mechanisms of deterioration are well understood, in order to inform conservation treatments, decision and policy making.

Given its status as a building material and how easily it can be damaged, it is of little surprise that limestone predominates in the current studies of biodeterioration, with sandstones and granites making up the majority of the other studies^4^. Analysis of the bacterial component of limestone and other calcium carbonate rock microbiomes is mainly approached from three directions in academic studies; the majority have looked at a sub-population of the microbiome from a biodeterioration perspective^4^, the role of microorganisms in the biogeological deposition of rock^7,8^, and the effect of climate and geochemistry on the microbiome^9,10^; while studies into primary and tertiary bioreceptivity^11–15^ are less common in the literature. Analysis of the microbiome is often limited to investigating damaged stone surfaces for biodeterioration and undamaged stone surfaces for biogeological studies. In the literature since 1997 the authors have found 61 papers directly addressing the microbiome, or components of it, associated with biodeterioration of limestone. Of these only 5^16–20^ (< 10%) had an undamaged control as a comparison, Antonelli et al.^19^ is a good example of this comparing patinated and unpatinated surfaces. This is not uncommon in the literature on biodeterioration of stone for example studies in granite^21,22^ and marble^22,23^ can easily be found which have not looked at undeteriorated surfaces. The focus for the 61 papers is mainly on damaged surfaces, with only a few making direct comparisons between the genera or species found on damaged and undamaged stone and as such it becomes more challenging to identify those microorganisms which are common to both. Of the 61 papers the authors identified only 3^24–26^ carried out species level testing to determine the role of isolates in biodeterioration. To fully understand the importance of the role that the bacterial microbiome plays in biodeterioration of stonework, all of the above aspects must be considered together. Climate has been shown to play a major role in the composition of the microbiome of carboniferous stone^9,22^ and in addition the geochemistry influences the particular species present^9,10^. With this in mind, any new studies investigating the role of microorganisms in biodeterioration must ensure that samples are taken from closely related stones exposed to the same climatic conditions. There is also conflict in the literature over whether species are significantly associated with damaged or undamaged surfaces and whether they play an important role in biodeterioration^27–32^.

The aim of this study is to characterise the differences in the bacterial component of the microbiome of Lincolnshire limestone found on damaged and undamaged stone, and to experimentally identify species which are actively involved in biodeterioration. In order to avoid the conflicts in previous studies mentioned above, this study is specifically designed to compare paired samples of Lincolnshire limestone from 4 distinct locations.

This study is the first to investigate paired sampling of physically damaged and undamaged stone, in a marine (Köppen–Geiger^33^ Cfb) climate, using metagenomics and as such clarifies the role of phyla and species in the microbiome. In addition this study is the largest to date for experimental characterisation of bacteria for biodeterioration processes and the first to show experimentally that the level of biofilm production on damaged surfaces is significantly higher than undamaged surfaces.

## Results

To confirm that the physical characteristics of the sampled stones were not affected by site, aspect or external environmental factors, and that differences between damaged and undamaged stone were only due to surface colonisation, the environmental data was analysed (Table 1). Significant (p-value < 0.05) correlations were found between the data which was not related to the stone surface (Supplementary Table 4), for example between relative humidity and temperature, relative humidity and lux, relative humidity and UV. Variation in temperature, relative humidity and light, both lux and UV, was related to the time and weather conditions when the sample was taken. There were no significant correlations between the environmental factors or aspect, and the surface wetness or surface pH for the sampled stone. Surface wetness on damaged surfaces gave a mean of 19 WMC, and a mean of 13 WMC for undamaged. A student’s t-test showed that there was no significant (p-value = 0.13) difference for wetness between damaged and undamaged stone. This supports the initial assumption that having sampling sites adjacent to each other would reduce the likelihood that differences between adjacent damaged and undamaged stone were caused by external factors.

**Table 1:** Environmental and surface measurements taken at each site.

Site	Aspect	Relative Humidity (%)	Temperature (°C)	Light (lx)	UV (µW/lumen)	Protimeter (WME)		Surface pH	
						Damaged	Undamaged	Damaged	Undamaged
Lincoln Cathedral	NE	83.4	12.2	500	800	18	15	5.5	6
Saint Peter-at-Gowts, Lincoln	SSW	80	8.5	4700	800	18	18	4.5	6
Burton Pedwardine	WSW	72.8	17.1	9324	1273	24	12	5	6
Saint Botolphs-by-Bargate, Lincoln	N	50.6	26.4	58,834	4	16	10	5.5	6

Environmental measurements were relative humidity, temperature, light and UV. Surface measurements were surface dampness using a protimeter, and surface pH, data shown is the mean for sampling where n > 3.

Analysis of pH of the stone surface gave a mean of 5.125 for damaged stone surfaces and a mean of 6 for undamaged stone, which was significant (students t-test, p-value = 0.035).

### Metagenomic analysis of the limestone microbiome

To assess the total bacterial microbiome, something which is not possible with traditional culture techniques as many species are not currently culturable in the laboratory, analysis of the metagenomic data was carried out at the phylum level of the taxonomy^29,34,35^. This requires a lower percentage identity than species level and allows the use of the full data set.

The 8 samples resulted in greater than 8 million raw reads which resulted in greater than 2.7 million paired end reads. Contig assembly resulted in greater than 48,000 contigs which were binned based on a coverage of > 120 × as per Zhang et al.^36^ with coverage being calculated using the Lander/Waterman equation^37^ as per Illumina guidelines^38^. This resulted in a final total of 2997 contigs which were carried forward for OTU & sequence level analysis. Sequencing data analysis, including a breakdown by samples, is available in Supplementary Table 2. All reads at this stage were longer than 200nt and had > 98% pairwise identity.

Coverage of the microbiome for each sample was calculated according to Good’s coverage estimator as was satisfactory (> 0.8)^20,39,40^ (Supplementary Table 2) especially as high diversity populations such as stone and soil require coverage > 0.5^41^.

Raw data from this study can be accessed via the NCBI sequence read archive as bioproject PRJNA775958 : Lincolnshire Limestone Metagenome, sample accession numbers are SAMN22636278 (St. Botolph-by_Bargate, damaged), SAMN22636279 (St. Botolph-by_Bargate, undamaged), SAMN22636280 (Burton Pedwardine church, damaged), SAMN22636281 (Burton Pedwardine church, undamaged), SAMN22636282 (Lincoln Cathedral, damaged), SAMN22636283 (Lincoln Cathedral, undamaged), SAMN22636284 (Saint Peter-at-Gowts, damaged) and SAMN22636285 (Saint Peter-at-Gowts, undamaged).

#### OTU level analysis of the stone microbiome

**Figure 1:**
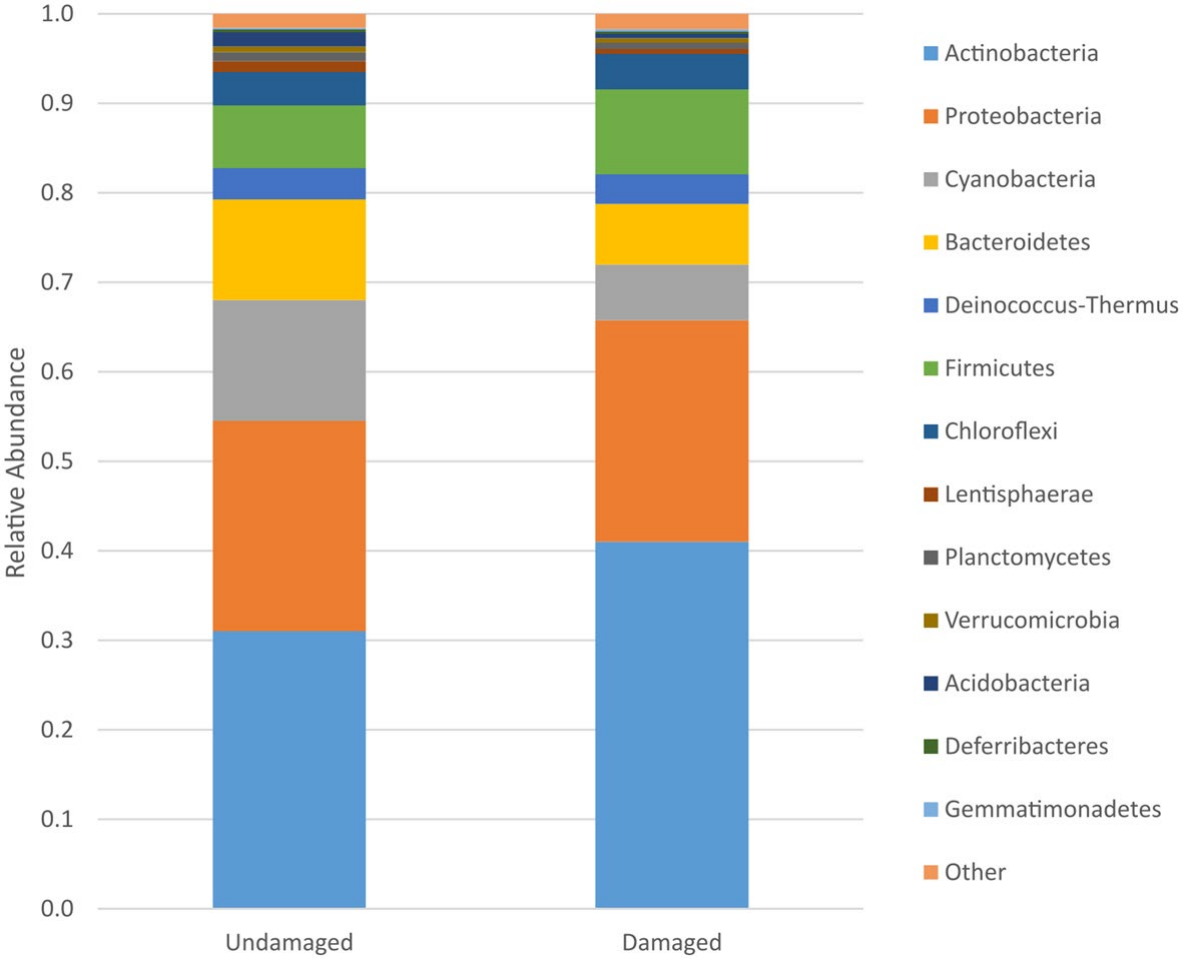
Phylum level taxonomy of damaged and undamaged stone communities based on the average 16S amplicon datasets of each community. While the 13 phyla shown compose more than 98% of these stone microbial communities the samples were clearly dominated by Actinobacteria, Proteobacteria and Cyanobacteria on both surfaces.

Overall composition of the stone microbial communities showed Actinobacteria, Proteobacteria and Cyanobacteria were the predominant phyla within the communities, collectively accounting for 68–77% of the total community (Fig. 1). The analysis sought significant associations between the microbiome and environmental conditions, aspect, light level and UV level and stone surface conditions. No correlation was observed between the different environmental/abiotic conditions when considering each individual monument and the microbial populations in damaged versus undamaged areas.

Bacterial phyla that were significantly more likely to be discovered on damaged surfaces were Actinobacteria, Proteobacteria, Firmicutes, Acidobacteria, TM7 and Thermotogae (p-value < 0.05). Bacterial phyla that were significantly more likely to be isolated from undamaged surfaces were Bacteroidetes, Cyanobacteria, Planctomyces, Armatimonadetes, Verrucomicrobia, Lentisphaerae, Sunergistetes, Caldiserica, Chlamydiae and Elusimicrobia (p-value < 0.05).

**Figure 2:**
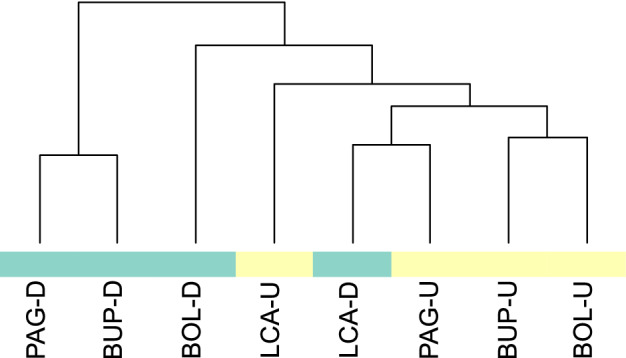
Distance matrix of metagenomic data analyzed at the OTU level. Analysis of the isolates at this level shows significant (p-value = 0.05) clustering according to whether the surfaces are: damaged—blue; or undamaged—yellow, with the exception of Lincoln Cathedral where the presence of Fusobacteria and Aquificae results in the clustering of the damaged sample with undamaged and the higher level of Acidobacteria grouping the undamaged site in with damaged sites.

The stone surface showed significant differences (p-value < 0.05) between the microbiomes of damaged and undamaged stone surfaces at the OTU level and to the species level of analysis. A distance matrix was generated using metagenomeSeq to visualise the relationship between the microbiomes present in the samples (Fig. 2). Samples which were from undamaged surfaces clustered together, showing less distance from each other than from the samples from damaged surfaces. Damaged surfaces were also primarily closer to each other than to undamaged surfaces. The only exception to this was at the OTU level with LCA-U and LCA-D showing a significant distance from each other but clustering LCA-D with undamaged and LCA-U with damaged. This was due to the presence of Fusobacteria and Aquificae (data not shown) in the damaged sample but not the undamaged sample; neither of these classes were significantly associated with damaged or undamaged stone. The distance matrix proves that there are significant differences between damaged and undamaged stone at each site and across all the sites.

### Identification of species from metagenomic sequencing data

While the total bacterial microbiome cannot be identified to the species level using the V4-V5 variable regions of the 16S rRNA genes it is possible to identify a proportion of the microbiome, between 50 and 80%^42,43^, to this level by applying suitable stringency^42–44^. In doing so this study has identified species which belong to the core microbiome, i.e. are specific to the microbiomes of different surfaces and provides a data set which can be compared against studies in the current literature.

The species were identified from the binned metagenomic contigs by searching against the NCBI prokaryote 16S rRNA database with > 97% sequence identity filter^45^. These results were then filtered based on e-value, sequence coverage of the V4–V5 region and pairwise identity with rejection criteria based on the current literature^42–44,46^. The search resulted in the identification of 1048 named species with 100% coverage of the variable regions, 99% pairwise identity and an e-value of 1.24 × 10^–3^ or lower.

The species identified were then assessed based on the number of sample sites they were isolated from and the surface they were sampled from (Supplementary Table 1). 59 of these were found in > 75% of the sites sampled forming a core microbiome; of the 59 species 68% were Actinobacteria, Proteobacteria or Cyanobacteria matching the OTU level analysis. The remaining 32% was split between Firmicutes (25.3%) and Bacteroidetes (6.7%) which were the next highest phyla in the OTU analysis. The core species made up 6% of those identified. The majority of species identified, 73%, were present in < 25% of the sites sampled indicating a strong geographical component to the microbiomes.

To confirm that a healthy, undisturbed population had been sampled^47^, Shannon’s index of diversity, Shannon’s equitability and Shannon’s exponential were calculated for each sample (Table 2). All 8 samples were shown to be representative of even sampling, Shannon’s equitability results were all > 0.8, from a healthy undisturbed population, Shannon’s index of diversity results were all > 3. As the bacterial census, and therefore the NCBI database, is currently incomplete^48^, and only a proportion of the population can be identified with the V4–V5 variable regions, Shannon’s exponential, estimation of the total population, was higher than the total number of species identified in each sample. Shannon’s exponential also showed that there was no significant difference between the total number of species found on either surface (p-value = 0.114).

**Table 2:** Metagenomics counts and analysis of Shannon's index of diversity, equitability and exponential results together with the percentage of species identified out of the total population estimated by Shannon’s exponential.

Sample code	Total contigs	Total isolates	Isolates at > 97%	Shannon’s Diversity Index	Shannon’s Equitability	Status of Ecosystem	Shannon’s Exponential	% identified
LCA-D	6864	700	376	7.21	1	Good	1352.63	27.80
LCA-U	7044	608	302	6.52	1	Good	678.92	44.48
PAG-D	2661	565	287	7.23	1	Good	1374.65	20.88
PAG-U	6110	694	375	7.21	1	Good	1347.27	27.83
BOL-D	11,441	2154	803	7.99	1	Good	2947.90	27.24
BOL-U	5111	503	178	5.23	1	Good	186.71	95.33
BUP-D	3961	634	313	7.96	1	Good	2859.29	10.95
BUP-U	6672	740	299	5.72	1	Good	305.74	97.80

As all the Shannon’s diversity index results are above 3 they represent a good ecosystem status. Shannon’s Equitability of 0.8 or above demonstrates even sampling across the species in the total population. Shannon’s Exponential gives an estimate of the total population size allowing the number of species identified to be estimated based on this and the number of isolates characterised at > 97% identity. Sampling codes are three letters for the location, number for the site and D or U for damaged or undamaged surface. LCA is Lincoln Cathedral, PAG is Saint Peter-at-Gowts, BOL is Saint Botolphs-by-Bargate and BUP is St. Nicholas, St. Andrew and the Blessed Virgin Mary's Church, Burton Pedwardine.

Significant differences between the species on the damaged surfaces compared to the undamaged surfaces were found. As with the OTU level of analysis significant (p-value = 0.05) differences occurred between the species found on the surfaces and when plotted as a distance matrix the samples to clustered with each other based on whether they were sampled from damaged or undamaged surfaces. Further analysis showed no significant clustering of samples was observed between species sampled and aspect, light level or UV level.

Three statistical tests showed (a) which of the species were most significantly (p-value ≤ 0.05) associated with the surface (log normal permutation test), (b) which of these species were most likely to be isolated during sampling (p-value < 0.05, Discovery odds test), and (c) presence-absence testing to identify whether these species were solely associated with the surface as opposed to being present at significantly higher levels on one surface or the other.

Eleven species were identified using log normal permutation as the top species associated with damaged stone, a further 14 were identified as associated with undamaged stone (Table 3). Of these species, discovery odds testing combined with presence-absence testing identified *Bacillus licheniformis* and *Rhodococcus sp.320* as being solely present on damaged surfaces and highly likely to be isolated during sampling. No species were identified as being solely present on undamaged surfaces using these tests.

**Table 3:** Species identified through log normal permutation as being significantly associated with damaged or undamaged surfaces.

Damaged	Undamaged
*Bacillus licheniformis*	*Bacillus pumilis*
*Bifidobacterium longum*	*Balneimonas flocculans*
*Brevundimonas *sp. *0312MAR21U9*	*Blastococcus ginsenosidimutans*
*Friedmanniella sagamiharensis*	*Calothrix *sp.* PCC*
*Gemella morbillorum*	*Gemmata *sp.* Br1-2*
*Gordonibacter *sp.* S475*	*Hyphomicrobium *sp.* ColF*
*Haemophilus influenza*	*Kingella oralis*
*Kineococcus bacterium*	*Kocuria rhizophila*
*Peptoniphilus *sp.* EL1*	*Pedobacter panaciterrae*
*Rhodococcus *sp.* 320*	*Phenylobacterium aquaticus*
*Shigella coli*	*Pseudonocardia seranimata*
	*Rothia mucilaginosa*
	*Solirubrobacter ginsenosidimutans*
	*Sphingomonas *sp.* DUSK*

### Characterisation of isolated bacteria for biodeterioration

A total of 70 separate isolates were cultured from damaged and undamaged surfaces (Table 4). Of the 70 isolates, 64 were unique species, the remaining 6 were strain variants of these species. The strain variants were *B. licheniformis* (2), *B. muralis* (3), *B. pumilis* (2)*, B. safensis* (2) and *B. subtilis* (2). Isolates were identified by sequencing their 16S rRNA genes except for *Microbacteriaceae* sp. *PAG4D,* which was confirmed to family level with the closest matches being 93%, as 97% is considered the cut off for species level identification^45^. This means that the isolate is most likely a new species. All of the isolates were then profiled for biofilm production and capacity for biocorrosion. Of these *Acinetobacter lwoffii, Arthrobacter agilis*, *Micrococcus luteus* and *Pseudomonas fluorescens* were identified as being part of the core microbiome, and as such were identified on all damaged and undamaged surfaces.

**Table 4:** Species identified through direct sampling analysed according to the environment they are isolated from adjusted in accordance with the sampling data from the metagenomics analysis.

Damaged	Both	Undamaged
*Acinetobacter johnsonii*	*Acinetobacter baylyi*	*Advenella kashmirensis*
*Arthrobacter protophormiae*	*Acinetobacter calcoaceticus*	*Arthrobacter phenanthrenivorans*
*Bacillus aerophilus*	*Acinetobacter lwoffii*	*Bacillus cecembensis*
*Bacillus foraminis*	*Arthrobacter agilis*	*Bacillus* sp. *BC11*
*Bacillus infantis*	*Bacillus cereus*	*Bacillus* sp. *PVS08*
*Bacillus licheniformis* strain *LCA3A7*	*Bacillus mycoides*	*Bacillus thuringiensis*
*Bacillus licheniformis* strain *PAG2D*	*Bacillus pumilis* strain *BUP2*	*Microbacterium ginsengisoli*
*Bacillus muralis* strain *LCA1D6a*	*Bacillus pumilis* strain *PAG4D2*	*Paenibacillus polymyxa*
*Bacillus muralis* strain *BUP3*	*Bacillus safensis* strain *LCA4*	*Pseudomonas brenneri*
*Bacillus muralis* strain *BOL1*	*Bacillus safensis* strain *BUP1*	*Psychrobacter faecalis*
*Bacillus niacin*	*Bacillus simplex*	*Solibacillus silvestris*
*Bacillus psychrosaccharolyticus*	*Bacillus subtilis* strain *LCA1D9*	*Sphingobacterium anhuiense*
*Bacillus sporothermodurans*	*Bacillus subtilis* strain *LCA1U3*	*Spongiibacter* sp. *IMCC21906*
*Brevibacillus brevis*	*Curtobacterium flaccumfaciens*	
*Isoptericola variabilis*	*Enterococcus hirae*	
*Lysinibacillus fusiformis*	*Escherichia coli*	
*Lysinibacillus parviboronicapiens*	*Exiguobacterium sibiricum*	
*Microbacteriaceae* sp. *PAG4D*	*Micrococcus halobius*	
*Microbacterium pseudoresistens*	*Micrococcus luteus*	
*Microbacterium schleiferi*	*Micrococcus roseus*	
*Microbacterium thalassium*	*Paenibacillus* sp*.1105*	
*Paenibacillus lactis*	*Pseudomonas fluorescens*	
*Paenibacillus lautus*	*Pseudomonas putida*	
*Paenibacillus pabuli*	*Pseudomonas stutzeri*	
*Pseudomonas brassicacearum*	*Sphingobacterium faecium*	
*Pseudomonas* sp. HZ06	*Sporosarcina saromensis*	
*Psychrobacillus psychrodurans*	*Staphylococcus xylosus*	
*Streptomyces microflavus*	*Stentrophomonas maltophilia*	
	*Stentrophomonas rhizophila*	

The additional data from the metagenomic sampling demonstrates that the majority of species isolated from undamaged surfaces were not specific to that environment. The damaged surfaces having double the number of specific species isolated when compared to the undamaged surface.

#### Biofilm formation

**Figure 3:**
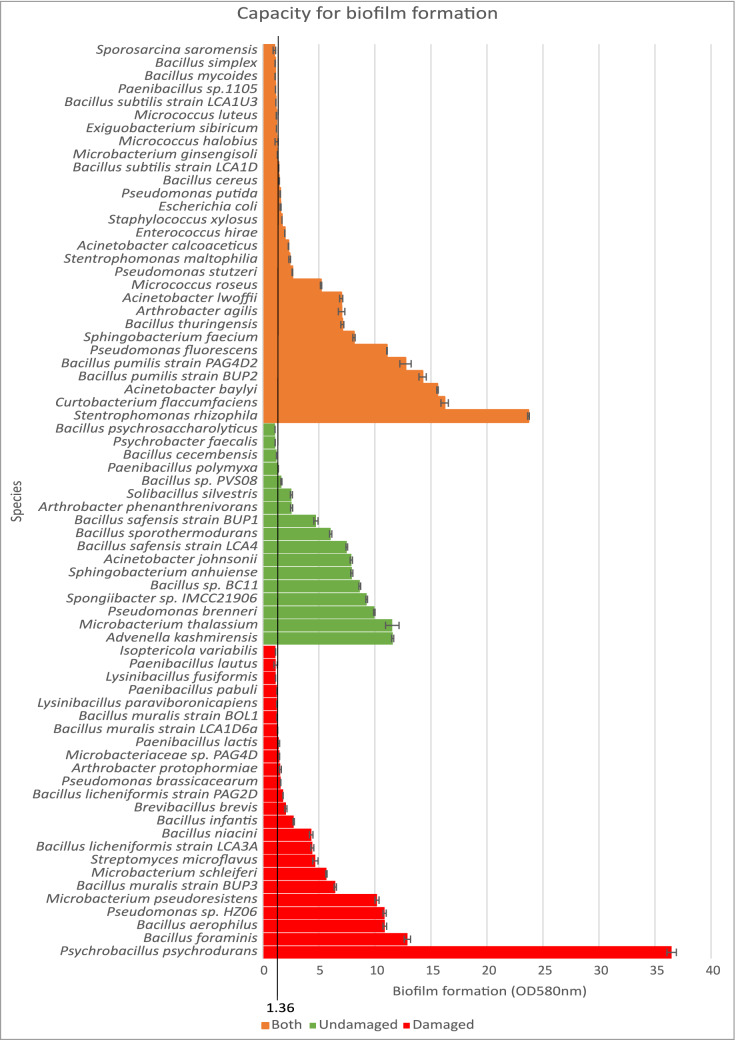
Biofilm formation capacity as a ratio of the OD580nm readings of the biofilm coated peg and the negative control peg. Data shown is average of n = 3. Species below 1.36 are poor or non-biofilm formers. A positive skew towards high biofilm formers is present in the isolates from damaged surfaces (red) as well as those isolates from both surfaces (orange). Isolates from the undamaged surface (green) demonstrate a normal distribution in their ability to form biofilms.

The presence of biofilm has been shown to enhance physical weathering of the limestone surface^5^. Therefore, all culturable species were assessed for biofilm formation by growing the individual isolates using the “Calgary peg” plate system with an adjusted ratio of biofilm formation: negative control to allow for direct comparison between plates. Biofilm formation ratios ranged from 36.49 for *P. psychrodurans* to 1.02 for *S. saromensis* which was considered not to produce biofilm under the test conditions, the biofilm OD reading being less than 1 standard deviation from the control. All other isolates which gave a low ratio had OD readings which were distinctly separate from the controls by two standard deviations (n = 3) and were therefore producing measurable amounts of biofilm. Species which produced a ratio below 1.36 for biofilm formation, cut off marked on Fig. 3, were considered as poor or non-biofilm forming. The cut-off point was calculated by taking the average of the lower quartile of the complete data set plus two times the standard deviation as per Croes^49^. Normality testing of the results was carried out and confirmed that the isolates from both damaged and undamaged surfaces were representative of the range of biofilm formation capability in the microbiome (p-value < 0.01).

As previously published studies have not used isolates from undamaged surfaces, no comparisons have been made between the capacity for species to form biofilm between the damaged and undamaged populations. In order to determine whether there was a significant difference in biofilm formation between the isolates from the two surfaces, the distribution of rates of biofilm formation was tested for skew. Isolates from damaged surfaces showed a strong skew towards high biofilm formation (G_1_ = 2.59, p-value = 0.05), whereas undamaged surfaces showed a lesser skew towards high biofilm formation (G_1_ = 1.37, p-value = 0.05). To determine whether the isolates which were found on both surfaces were responsible for the undamaged population demonstrating a skew towards high biofilm formers the data set was split in 3, looking at isolates associated with damaged, undamaged and both surfaces, and retested. The population isolated solely from undamaged stone showed no significant variation from a normal distribution. The isolates which were found on both surfaces gave a skew of G_1_ = 1.55, p-value = 0.05, demonstrating a skew towards high biofilm formers. In isolates solely from damaged stone, G_1_ = 3.29, p-value = 0.05, the skew in the data set was influenced by the extremely strong biofilm production of *P. psychrodurans*, removing this result from the data set reduced the figure to G_1_ = 1.3, p-value = 0.05, which was still a significant skew towards high biofilm formers.

This study has therefore shown for the first time that the bacterial population found as part of the limestone microbiome on damaged surfaces is significantly skewed towards high biofilm formers, unlike the population found solely on undamaged stone.

#### Biocorrosion

In the literature to date two approaches have mainly been used to investigate biocorrosion, testing for dissolution of calcium carbonate in an agar based medium^50^, and direct culturing on limestone blocks followed by direct observation using scanning electron microscopy (SEM)^51^. Both these techniques were used here in order to characterise biocorrosion, with dissolution in agar used as an initial screening approach to select bacteria for further investigation using SEM. Dissolution of calcium carbonate by isolates was observed as an endpoint experiment using solid growth media, which is made opaque by the presence of insoluble calcium carbonate. Dissolution results in first the colony becoming more translucent and then a zone of translucency around the colony (Table 5).

**Table 5:** Species demonstrating measurable dissolution of agar suspended Calcium carbonate after a period of 15 days.

Species	Clearance zone (mm)
*Bacillus sporothermodurans*	8
*Pseudomonas brenneri*	4
*Solibacillus silvestris*	4
*Spongiibacter *sp. IMCC21906	7
*Acinetobacter calcoaceticus*	12
*Arthrobacter agilis*	14
*Bacillus cereus*	3
*Bacillus safensis* strain LCATB3	7
*Micrococcus luteus*	2
*Micrococcus roseus*	10
*Pseudomonas putida*	10
*Pseudomonas stutzeri*	4
*Staphylococcus xylosus*	8
*Stentrophomonas maltophilia*	6

Of the 70 isolates tested, fourteen species were identified as actively dissolving calcium carbonate in this study, *Bacillus sporothermodurans* from the damaged stone population, *Pseudomonas brenneri*, *Solibacillus silvestris*, and Spongiibacter sp. IMCC21806 from the undamaged population and *Acinetobacter calcoaceticus*, *Arthrobacter agilis*, *Bacillus cereus*, *Bacillus safensis* strain LCATB3, *Micrococcus luteus*, *Micrococcus roseus*, *Pseudomonas putida*, *Pseudomonas stutzeri*, *Staphylococcus xylosus* and *Stentrophomonas maltophilia* were from the isolates found on both surfaces. *P. putida* showed the strongest dissolution with a clear 10 mm diameter halo around the colonies within 7 days. The remaining 12 species took 3 weeks to develop identifiable zones of dissolution, ranging from 6-12 mm diameter around the colonies. *Micrococcus luteus* was the weakest showing translucency under the colony with 1 mm diameter visible clearance around the colonies.

**Figure 4:**
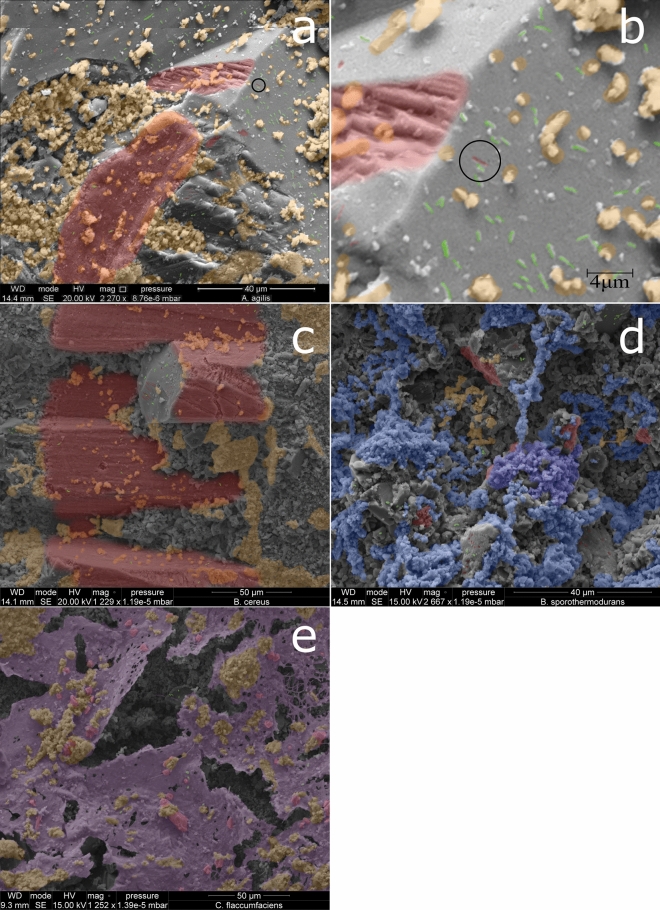
SEM images showing biofilm presence and areas of pitting and etching. Biofilm is shaded orange unless calcified when it is blue or a biofilm sheet when it is violet. Bacterial cells are shaded green when not encapsulated in biofilm matrix. Areas of damage are shaded red. Images (**a**, **b**) Black ring on Arthrobacter agilis picture highlights a very good example of bacterial pitting. Image b shows an enlarged copy of the area within the ring, the bacterial pit is highlighted in red and is the same dimensions as the surrounding cells, highlighted in green. (**c**) SEM image of Bacillus cereus showing high levels of etching on nailspar crystals, red, the crystal surface is naturally smooth making it easy to identify the pitting (holes in the surface) and etching (long grooves in the surface). Biofilm is shaded orange. Bacterial cells are shaded green when not encapsulated in biofilm matrix. (**d**) Bacillus sporothermodurans demonstrated high levels of calcification of the biofilm matrix, blue. Uncalcified biofilm is shaded orange with pitting and etching shaded red. Bacterial cells are shaded green when not encapsulated in biofilm matrix. (**e**) Curtobacterium flaccumfaciens produced two forms of biofilm matrix, sheets and clusters. The sheets showed evidence of physical damage caused by the biofilm matrix, shaded purple, growing through the limestone oolitic matrix and lifting the smaller oolites, shaded red, from the surface. Where the biofilm formed in clusters it is shaded orange, bacterial cells are shaded green when not encapsulated in biofilm matrix.

By testing for biocorrosion through direct observation in a different growth environment the potential for growth media to alter metabolic pathways and suppress acid production can be partially overcome. 24 culturable species from the damaged population along with 13 species which had tested positive for calcium carbonate dissolution from the other two populations, as controls, were selected for testing. All species inoculated onto the limestone blocks in the laboratory showed evidence of biofilm formation on the surfaces (Fig. 4). Images showed micro pitting and etching of the surface and compared with SEM images of known examples from the literature^11,28,32,51–56^. Four modes of bacterial damage were observed, including a new form of damage not previously characterised in the literature. Micro pitting, where individual cells have dissolved the surface was visible at a bacterial scale with some surfaces showing evidence of bacterial etching, resulting in etched troughs in what should otherwise be smooth crystalline surfaces. An example of this is highlighted in the *A. agilis* SEM image, Fig. 4a, with a black circle surrounding the pit, false coloured red, which is the exact dimension of the surrounding cells. This section of the picture has been enlarged for clarity (Fig. 4b). Evidence of bacterial etching of the surface, areas false coloured in red were identified on nailhead spar crystals of calcium carbonate which have completely smooth faces when uncorroded. These are the crystals with hexagonal cross sections seen in the SEM image of *B. cereus* (Fig. 4c).

*B. sporothermodurans* was one of the species which showed evidence of calcification of the biofilm matrix, false coloured blue, Fig. 4d. Calcification was identified from structures which matched those shown in the literature as calcified biofilm, or biofilm structures which matched the literature but were also encrusted. A particularly good example of this is the calcified biofilm string in the top centre of the *B. sporothermodurans* image. Levels of calcification varied between species, for *S. maltophilia* and *S. silvestris* only calcified biofilm was observed where for the other species calcification appeared to be present only in mature biofilms. Finally both *C. flaccumfaciens* (Fig. 4e) and *S. silvestris* showed evidence of physical damage with the biofilm matrix growing through the limestone oolite and lifting them from the surfaces. Both of these species produce a biofilm sheet, false coloured violet in the SEM image, which was not observed in other species, as well as the globular biofilm matrix which is found elsewhere. The small oolites which have been removed from the surface by the *C. flaccumfaciens* biofilm have been false coloured red to distinguish them from the biofilm sheet. This mode of physical damage is not one which has been previously described.

With the calcium carbonate media tests and the SEM observations combined, 18 species demonstrated potential for biocorrosion, 6 species were identified as causing calcification and 2 species demonstrated the novel mechanism of biomechanical weathering. These are identified in the summary table (Table 6).

**Table 6:** Summary of the properties of species associated with biodeterioration and biocorrosion, together with their prevalence across the sample sites.

Surface	Species	Identified in the literature	Prevalence	Physical weathering	Biomechanical weathering	Biochemical modification	Biocorrosion
			% sites sampled	Biofilm	Biofilm	Calcification	
Both	*Arthrobacter agilis*	^29,31,82^	100				X
	*Micrococcus luteus*	^24,29,31,64^	100				X
	*Pseudomonas fluorescens*	^67^	87	X			
	*Acinetobacter calcoaceticus*		62			X	X
	*Pseudomonas stutzeri*	^29,66^	37				X
	*Bacillus cereus*	^28,53,63,66,83,84^	25				X
	*Curtobacterium flaccumfaciens*	^29^	25	X	X		
	*Sphingobacterium faecium*		25	X			
	*Stentrophomonas maltophilia*	^7,64^	25			X	X
	*Micrococcus roseus*	^53,82^	13				X
	*Acinetobacter baylyi*		12	X			
	*Bacillus safensis*		12			X	X
	*Pseudomonas putida*	^29,85^	12				X
	*Staphylococcus xylosis*		12				X
	*Stentrophomonas rhizophila*		12	X			
	*Bacillus pumilis*	^7,63,64^	6	X			
	*Bacillus thuringensis*	^32,64^	6	X			
Undamaged	*Advenella kashmirensis*		25	X			
	*Pseudomonas brenneri*		25	X			X
	*Solibacillus silvestris*		25		X	X	X
	*Microbacterium thalassium*		6	X			
	*Spongiibacter* sp*. IMCC21906*		6	X		X	X
Damaged	*Bacillus licheniformis*	^29,31,63,86^	100				X
	*Bacillus muralis*		50	X			X
	*Psychrobacillus psychrodurans*		50	X			
	*Bacillus aerophilus*		25	X			
	*Bacillus sporothermodurans*	^31^	25			X	X
	*Paenibacillus lautus*		25				X
	*Bacillus foraminis*		6	X			
	*Microbacteriaceae* sp. *PAG4D*		6				X
	*Microbacterium pseudoresistens*		6	X			
	*Pseudomonas* sp*. HZ06*		6	X			

Species which are shown as enhancing physical weathering through biofilm production all produced high levels of matrix. Biophysical weathering through biofilm identifies the species where the biofilm matrix was disrupting the stone structure as opposed to enhancing physical weathering.

## Discussion

This is the only study to date to have analysed the marine (Cfb) limestone microbiome on both damaged and undamaged surfaces across multiple sites, investigating the bacterial components of the core microbiome in addition to associations of species between physically damaged and undamaged areas. The study seeks to resolve conflicts in published literature as to whether species or phyla are more strongly associated with one type of surface. By ensuring the locations are geographically distinct while maintaining a common climate and geochemistry, any species which were common to all samples would be regarded as the core microbiome for Lincolnshire limestone as opposed to being specific to one site. For example studies such as Schröer et al.^25^, which looks at damaged Lede limestone in a Cfb climate, identified many species in common with this study such as *A. agilis* and *A. tumbae* which we are able to characterise as species common to both damaged and undamaged surfaces, as well as *P. psychrosurans* and *C. flaccumfaciens,* both of which have been demonstrated in this study to potentially enhance damage through high levels of biofilm formation. Differences between the species identified are therefore likely to relate to geochemistry of the stone^9,10^ and pollution^27^.

The approach to the investigation of the microbiome varies between worldwide studies. While the majority of the studies attempt to identify the isolates to species level, many studies only revealed the level of genus or class^27,28,57,58^ which is commonly used in metagenomics studies where it is described as an Operational Taxonomic Unit (OTU)^59^. Some studies do not determine species or class of organism is present in the sampled population, and some just state that bacteria are present without carrying out identification^34,60,61^. While these studies help to provide a broader understanding of the microbiome, their limitation is that on their own they cannot identify the species involved in biodeterioration nor the mechanisms thereof. Ding et al.^62^ addresses this issue stating that while next generation sequencing is important to gain an overview of the microbiome it should be supported by culture dependent and independent methodologies to both advance scientific research on cultural heritage and provide evidence to inform the protection and management of heritage materials.

Biodeterioration studies at the species level focused on damaged stone. Some common species, *Bacillus cereus, Bacillus mycoides, Bacillus licheniformis, Bacillus pumilis, Bacillus subtilis, Micrococcus luteus, Pseudomonas putida,* and *Stentrophomonas maltophilia* were all identified in 3 or more papers^28,32,53,63,64^. However, the contribution of these species to damage is unclear, as the results from studies are often contradictory due to the ongoing debate between the role of biocorrosion and bioprotection mechanisms, and the context of the study. For example Di Bonaventura et al*.*^50^ and Saikia^65^ identify *Bacillus licheniformis* as a producer of oxalic acid and suggest that its presence on a calcium carbonate based stone could be protective due to the formation of a calcium oxalate coating whereas Nuhoglu et al*.*^32^ identify *B. licheniformis* as potentially damaging due to the production of biocorrosive acids including oxalic acid.

This study has, for the first time, allowed an accurate representation of the microbiome from damaged stone by identifying which of the species identified in past studies are not unique to it. There were significant differences in the bacterial microbiomes present on damaged and undamaged limestone at the OTU level which was reflected in identical analysis of the isolated culturable bacteria and those which were able to be identified from the metagenomic sequence data. The core phyla identified in the OTU level data align with Mediterranean, African and Asian studies^9,34,57^, these studies looked primarily at undamaged stone, showing that the differences shown between the microbiomes of damaged and undamaged limestone at the phyla level are most likely applicable internationally. Both species identification from the metagenomic sequences and direct sampling were comparable including the isolation of *B. licheniformis* and *B. aerophilus* solely from damaged surfaces. Several species, such as *M. luteus, P.* sp.*1105* and *P. psychrodurans,* while found on both surfaces, were significantly more likely to be isolated from damaged stone which is supported by their discovery on damaged surfaces in studies such as Flores et al.^53^, Schröer et al.^25^ and De leo et al.^29^. The data from this present analysis has been made available as supplementary data to support future studies. At the OTU level there were also statistically significant differences between the two surfaces.

The species identification results here help to explain some potentially conflicting published results. *B. thuringensis*, *P. polymyxa*, *P. fluorescens*, *P. stutzeri* and *S. maltophilia* were all isolated from both damaged and undamaged limestone surfaces in this present study, however previously published literature^4,7,29,31,32,64,66,67^ stated that they were significantly associated with damaged surfaces. The species identification carried out from the metagenomic data showed that they were associated with both environments, in fact *P. fluorescens* is part of the core microbiome, although within the scope of this study it was not possible to state whether the other species were significantly associated with either surface. The fact that the species level analysis of the damaged and undamaged microbiomes clarifies previous conflicts in the literature, where the studies were carried out across a range of environments and different limestones, supports the identification of the core species as being internationally relevant with the rest of the species identified being more specific to the climate and geochemical differences of the substrate.

Many studies have claimed that *Bacillus* species are associated with damaged surfaces while others stated a significant association with undamaged surfaces^7,27,29,30,50,68^. In this present study, by comparing damaged and undamaged surfaces, it has been possible to demonstrate that many of the *Bacillus* species identified showed no significant preference for damaged stone and were found on both surfaces. Indeed, some of these *Bacillus* species make up the core of the microbiome.

This study adds significant new understanding to the biodeteriorative mechanism of stone deterioration, through demonstrating the clear link between the bacteria isolated from stone and their deteriorative process. We detected four mechanisms of biodeterioration during the testing of the isolates, enhanced physical and geochemical weathering of the stone through the presence of high levels of biofilm matrix, biocorrosion, biochemical modification of the stone matrix and biomechanical weathering of the stone by biofilm matrix. Half (32) of the species identified in this study demonstrated biodeteriorative capacity through one or more of these mechanisms; 19 of the species demonstrating biodeteriorative capacity had not been previously identified in the literature as being colonisers of the stone surface (Table 6). As the species identified in the literature have been found in different climates and on limestones of differing geochemistry the experimental evidence of biodeterioration of these species is applicable internationally. The significantly lower surface pH of damaged stones is likely to be related to additional biodeteriorative processes via secretion of organic acids.

Of the 64 species tested only *Sporosarcina saromensis* was classified as not producing biofilm, therefore 98.5% of the culturable species were biofilm formers. Eighteen of the isolates were classed as weak biofilm formers, due to strain to strain variation in biofilm formation this works out at 25% of species tested; these species are therefore unlikely to substantially contribute to the enhancement of physical weathering through biofilm formation. The species which are the strongest biofilm formers (Table 6) are most likely to rapidly enhance physical weathering due biofilm secretion; the key contributors identified in this study being *P. fluorescens, B. muralis* and *P. psychrodurans* based on their prevalence at the sampling sites and the high levels of biofilm formed. The analysis of biofilm formation by species, based on the surface they were sampled from, provides a direct link between species which shows, for the first time, a higher production of biofilm matrix by species associated with damaged surfaces.

Species capable of biocorrosion (Table 6) made up a much higher proportion of the damaged population, and the population found on both surfaces, than the undamaged population.

Of the 18 species identified as dissolving calcium carbonate in this study, 8 of them, *A. calcoaceticus*, *B. safensis*, *M.* sp*. PAG4D, P. lautus*, *P. brenneri*, *S. silvestris*, *S.* sp. *IMCC21906* and *S. xylosus* had not been previously identified as damaging to the limestone surface. Three of the above species were only isolated from undamaged surfaces. The only isolates in this study which were isolated solely from a damaged surface which dissolved calcium carbonate were *Bacillus licheniformis* and *Microbacteriaceae* sp*. PAG4D*, a potential new species. This demonstrates that biodeteriorative bacterial species are not solely focused on damaged areas; instead a holistic sampling approach should be considered when evaluating the microbiome.

Only 3 of the species identified as strong biofilm formers were demonstrated to cause biocorrosion, *P. brenneri*, *S.* sp. *IMCC21906* and *B. muralis* (Table 6). It is possible that the remaining 15 species are using biocorrosion to engineer the environment into one which better fits their growth requirements.

Based on prevalence in the sampling *A. agilis*, *A. calcoaceticus*, *B. licheniformis*, *B. muralis*, *M. luteus* and *P. stutzeri* should all be considered key contributors to biocorrosion as they were present in 30% or more of the sites sampled. *B. licheniformis* and *B. muralis* were specific to damaged surfaces and found on 100% and 80% of damaged sites respectively. The other species are present on all surfaces but were found on 60% of damaged surfaces compared to 40% of undamaged.

While the sample numbers are too small to determine the significance it is interesting to note that of the 3 species identified as actively dissolving calcium carbonate on undamaged stone, two were also calcifying their biofilm matrix. When this is compared to the isolates found on both surfaces only 3 of the 10 isolates produced calcification, and only 1 of the 5 isolates which dissolved calcium carbonate on the damaged surface also calcified the environment. This suggests that the action of species which dissolve calcium carbonate on visibly undamaged surfaces could be more likely to result in re-deposition of the calcium carbonate. The dissolution and redeposition of the stone around the biofilm matrix will result in alteration of the physical characteristics of the stone surface; whether in this case it is harmful, with the flexible matrix resulting in a more friable surface, or beneficial as the deposited calcium carbonate will penetrate the stone surface in a similar fashion to the consolidating nanomaterials used in stone conservation, is beyond the scope of the current study.

One mechanism of biodeterioration identified in this study has not been previously identified. The biofilm matrix of *Curtobacterium flaccumfaciens* and *Solibacillus silvestris* was observed growing through the oolitic matrix of the limestone and physically detaching the oolites from the surface, a new mechanism of biomechanical weathering. Previously, the weathering caused by the biofilm matrix is only ever assumed to be caused by enhancing the effects of physical weathering, expansion and contraction of the matrix with changes in relative humidity and temperature accelerating the effect which these environmental changes would have on the stone structure. This adds significant new information to aid understanding of deterioration mechanisms of stone.

To conclude, this study provides insights for conservation professionals and heritage scientists into the microbiome of one of the most common building stones. Prior to this study the differences between the microbiome on damaged and undamaged surfaces was not fully investigated and there were several conflicts regarding the significance of particular species or phylum being found on these surfaces. We have shown that the microbiome present on damaged stone is significantly different to that on undamaged surfaces and that there is a core microbiome shared between the two. Species such as *B. licheniformis* have also been demonstrated to be specific to the damaged microbiome and experimentally proven to cause biodeterioration. Biofilm formation has also been demonstrated to be higher in species isolated from damaged surfaces, and a novel mechanism of bio mechanical damage via biofilm lifting off surface material identified.

Low surface pH and the presence of *B. licheniformis* have both been shown to be significantly associated with the biodeteriorative microbiome. These are both characteristics which would benefit from further investigation in order to determine whether they could be used as general markers to identify surfaces which are suffering from biodeterioration, for example pH monitoring could identify the shift from a non-damaging microbiological patina to active biodeterioration.

## Material and methods

### Sample sites

**Figure 5:**
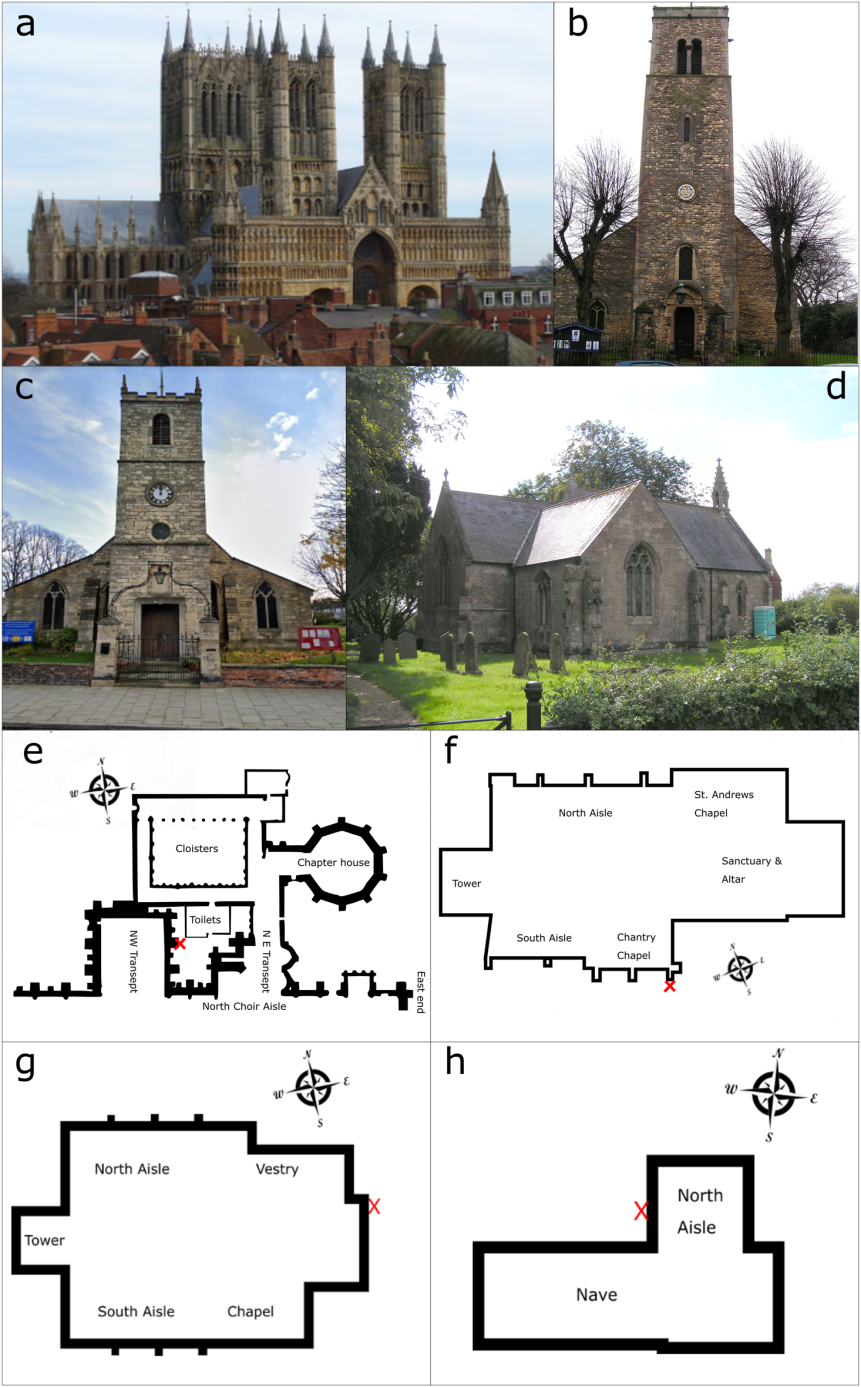
Images and maps of the sampling sites, maps are not to scale. Images (**a**, **e**) are respectively a picture and map of the north east section of Lincoln Cathedral with the sampling site marked by a red X on the map. Images (**b**, **f**) are respectively a picture and map of Saint Peter-at-Gowts with the sampling site marked by a red X on the map. Images (**c**, **g**) are respectively a picture and map of Saint Boltophs-by-Bargate with the sampling site marked by a red X on the map. Images (**d**, **h**) are respectively a picture and map of Saint Andrews Church, Burton Pedwardine with the sampling site marked by a red X on the map. All images were taken or drawn by Philip Skipper.

Samples were taken from adjacent damaged and undamaged external limestone building surfaces. Sampling was carried out at Lincoln Cathedral (LCA-U & LCA-D), Saint Peter-at-Gowts (PAG-U& PAG-D), Lincoln, Saint Botolph-by-Bargate (BOL-U & BOL-D), Lincoln, and Saint Nicholas, Saint Andrew and the Blessed Virgin Mary’s Church (BUP-U & BUP-D), Burton Pedwardine (Fig. 5). Permission was obtained from the Vicars of the churches before sampling was carried out. In addition, permission was granted by the Masters Committee of The Cathedral Church of the Blessed Virgin Mary of Lincoln (Lincoln Cathedral) to allow sampling to take place around the site. Where possible, sites were chosen where an undamaged stone was adjacent to a damaged stone with the same orientation and exposure, both being sampled in order to achieve a direct comparison of bacterial colonisation. Surfaces were considered physically damaged if spalling or fragmentation to a depth > 10 mm from the surface had occurred^69^. Samples were taken from external locations which, according to records, had not been cleaned or treated with biocide in order to ensure that the microbiome sampled was not influenced by past treatment of the surface.

### Surface sampling

Sampling of the stonework for culturing the bacteria, or extracting the total genomic DNA from the microbiome for metagenomics studies, was performed using sterile swabs^27^; this method was selected following an evaluation of different techniques, with swab sampling showing the highest recovery of culturable species.

To obtain samples a sterile swab was dipped in sterile M9 salts (M6030, Sigma) and wiped over a 1 cm square region of the stonework next to the sellotape sampling area. Fresh M9 salts were used for each sample to prevent cross contamination between sampling sites. At all stages of sampling nitrile gloves were worn to prevent contamination of the samples with skin microbiota.

### Environmental data recording

Relative humidity and light (lux and UV) measurements were taken at each recording site using an Elsec 765 Environmental Monitor (Littlemore Scientific). Moisture readings of the surface of each stone sampled were obtained using a protimeter (GE Protimeter Mini BLD2000). Surface pH readings were taken using narrow range pH paper (Whatman, pH 4–6 and 6–8) moistened with distilled water.

Information regarding details of the sampling location which could affect the nature of the biome such as guttering, vegetation proximity and water flow were recorded along with the direction the stone face (aspect), height from ground level and location on the wall. The latter data, along with photographs and a sketch of the sampling site, were taken to allow accurate resampling at a future date if necessary, sampling sheets are available as Supplementary Fig. 1.

### Culturing and isolation of bacteria

Micro-organisms were isolated from the swabs by adding 1 ml of M9 salts to the swab holder and vortexing at full speed for 5 min. The resulting suspension was then plated out onto non-selective media—Nutrient Agar (Oxoid CM0003B), and grown for 96 h at 25 °C in normal atmosphere. 25 °C was selected experimentally as the temperature at which the majority of species isolated from the initial sampling showed optimal growth under laboratory conditions. Plates were then inspected and where possible single bacterial colonies were selected and re-streaked onto Nutrient Agar based on variations in colour and morphology. The isolates were then grown for 96 h at 25 °C. On plates where the growth was confluent, samples were taken and re-streaked onto Nutrient Agar and grown for 96 h at 25 °C. This process was repeated until single colonies could be isolated. Following the isolation of single colonies on the plates they were then left for a further 96 h at 25 °C to confirm that there were no slower growing organisms, whose presence was obfuscated by the selected species. In cases where these were seen, the two species were separated and re-streaked, again being grown for the appropriate period at 25 °C.

### Identification of isolated species

Bacterial genomic DNA was extracted using an in-house protocol which is suitable for both Gram positive and Gram negative bacteria. Briefly, cultured cells were pelleted by centrifugation (17,000×*g* in a Hereus Pico 17 microcentrifuge). The supernatant was disposed of and the pellet resuspended in 100 µl of TE (Sigma, T9285-100ML) buffer with 2 µg of a 1 mg/ml stock Lysozyme (Sigma, 62971-10G-F) added then incubated, shaken, for 30 min at 37 °C. 50 µl of a 10% SDS stock was then added and the sample was incubated at 60 °C for 30 min. Insoluble material was pelleted by centrifugation and the supernatant transferred to a fresh tube. A standard phenol chloroform isoamyl alcohol extraction was performed with a final chloroform step to ensure the elimination of any phenol from the sample. DNA was precipitated with ice cold 70% ethanol and then re-solubilised in 30 µl of ultrapure water. Where it was not possible to use the extracted DNA in a PCR reaction immediately, samples were stored at − 20 °C.

Regions of the 16S rRNA gene were PCR amplified from the extracted genomic DNA for each isolated species. The amplification was performed twice, once with the commonly used Universal primers amplifying a 1498 bp region between nucleotides 27 and 1525^70^ and once with primers designed by Dr. Michael Shaw, University of Lincoln, which amplify a 322 bp region between nucleotides 764 and 1084 (Supplementary Table 3). The PCR program was run with an initial denaturing step of 95 °C for 10 min, followed by thirty-four cycles of 95 °C for 30 s, 47.9 °C for 30 s and 72 °C for 1 min 30 s. The run ended with a final extension step of 72 °C for 10 min. Amplification was performed using OneTaq® 2 × Master Mix (New England Biolabs, M0482). PCR products from both reactions were sent to Beckman Coulter Genomics or Durham Genomic Services for sequencing. Sequencing data was reviewed using Geneious version R9^71^ and then used to identify the individual species by BLAST searching the sequence on the NCBI website.

Where poor sequence was returned species were checked for multiple 16S rRNA genes using 2% agarose gels on the PCR product using PS_16S_F555 and MS_BACT-16S_Rev as primers (Supplementary Table 3) which covers variable regions 4–8. PCR products were then TOPO cloned (K457502, Invitrogen) and the plasmids amplified and extracted as per the manufacturers instructions prior to plasmid extraction (QIAGEN Mini-Prep plasmid extraction kit 27104, QIAGEN) and sent for sequencing.

### Metagenomics

Total genomic DNA was extracted from swabbed samples of stonework using the phenol chloroform protocol described in 2.5. The extracted DNA was given a final wash in 50 µl of 10 mM Tris (Sigma T6791), 5 mM EDTA (Sigma E6758) and 5 mM EGTA (Sigma E0396) then the ethanol precipitation step was repeated, to remove any remaining calcium ions which could interfere with the PCR amplification of the 16S genes. Samples were sent to Nottingham Trent Universities Genomics service to be run on an Illumina MiSeq next generation sequencing system using the Earth Microbiome Project primers^72^. These amplify between 515F/806R and give greater than eighty percent identity of bacterial and archaeal species.

Analysis was carried out using Geneious version R9^71^ following the metagenomic analysis workflow^73^ for each sample. In brief, reads were paired, trimmed to remove the illumina adapters and any bases below an average score of 30 then any reads which were less than 100 bp were removed. Paired end reads were then merged to produce a consensus sequence for each pair with chimeric reads being removed. A high stringency de-novo assembly was then carried out to cluster the contigs into OTU’s and then BLAST searched against a local copy of the ncbi 16S microbial database. These were then used to set up a local database for each sample which was analysed using the Geneious Sequence Classifier plugin and the 16S Biodiversity tool. Data outputs from these tools were then used for comparative analysis.

For the subset of the microbiome where species level identification was possible^42–44^ a BLAST search was carried out on the contigs with > 120 ×^36^ coverage with a filter of > 97% identity^45^. These results were then sorted by e-value and filtered to remove results with < 100% sequence coverage of the V4-V5 region and pairwise identity of less than 99%, this stringency levels were based on the current literature^42,43,46^.

Analysis of the data for the identified OTU’s and identified species were carried out using metagenomeSeq package for R^74^ as it is designed for datasets of this size, details of the tools used is further discussed in Sect. 5.10.

### Characterisation of biofilm formation

Each isolated species was cultured to 0.01 (OD 580 nm) as per the planktonic growth curves. Aliquots of 100 µl of each dilution for each species was placed into a 96 well plate and a Nunc Immuno TSP 96 peg lid (Fischer Scientific, 445497) was applied. The plates were sealed with parafilm (Sigma P7793) and incubated at 25 °C for 72 h to allow all of the species sufficient time to produce a mature biofilm^75^.

The mature biofilm was quantified by staining with crystal violet for 2 min followed by two ten min washes in an excess of deionized 18MΩ water. Crystal violet which was bound to the biofilm matrix was eluted in 100% ethanol and the eluate read on a BMG Fluostar Optima at 570 nm. All results were produced from the mean of three repeats.

### Effects of bacterial metabolism on the dissolution of calcium carbonate

Dissolution of calcium carbonate by bacterial metabolic by-products was tested on a solid agar medium as per the method of Di Bonaventura et al.^50^*,* with the following modifications. Calcium carbonate in the enriched medium was reduced from 50 to 20 g/L which resulted in a more homogenous dispersion of calcium carbonate through the medium. The additional supplementation of calcium carbonate for the carbonate solubilisation test was shown to be unnecessary. Glucose in the enriched medium was reduced from 67 to 1 g/L, in line with more commonly used growth media, after it was shown to inhibit growth of many species at the original concentration. All results were produced from the mean of three repeats.

### SEM analysis of monoculture biofilms

Isolates were cultured in a liquid form of the growth media described in Di Bonaventura et al.^50^, without the addition of calcium carbonate to the media. Instead a 1 cm^3^ sterile limestone cube was partially immersed in the media to provide a source of calcium carbonate and a surface for the bacteria to form a biofilm matrix on. The culture was grown statically at 25 °C for a 3 month period with growth media being refreshed under sterile conditions every two weeks.

All samples were prepared for viewing on the SEM by coating in 0.1 M sodium cacodylate (Sigma C0250), fixing in 3% glutaraldehyde (Sigma G7776), washed in deionized water then dehydrated by working through a series of 50, 70 and 100% methanol (Sigma 322415) then a final step of 100% acetone (Sigma 439126). The stone blocks were then air dried, attached to SEM stubs and splutter coated using a gold palladium electrode to reduce damage to the biofilms and earthed with silver conductive paint to prevent charge buildup^76^. Imaging was carried out with the FEI 2017/11 Quanta Inspect SEM throughout.

### Statistical analysis of data

Analysis of data was carried out using R, version 3.3.1, or where packages were not available for R using Microsoft Excel 2013.

Good’s coverage estimator was used to determine the coverage of the microbiome present in each sample and was calculated with the OTU data using Excel with the equations from the original paper^77^.

Shannon’s index of diversity and Shannon’s equitability were both calculated using Excel using the equations provided in the original paper^78^.

Microbiome analysis was carried out on the phyla identified from the metagenomic data and the species identified from the metagenomics sequences and directly sampled studies. This was achieved using 4 of the tools from the metagenomeSeq package in R^79^, which was selected as it is designed to handle smaller data sets and is specifically targeted at analysis of differences between microbial communities. The plotMRheatmap function of metagenomeSeq generates a tree to provide visualisation of statistically significant clustering. Log normal permutation testing determines whether species, or OTUs, are significantly present in one sample when compared to the other. Discovery odds ratio testing determines whether species, or OTUs, are significantly likely to be isolated during sampling. Finally presence-absence testing determines whether species, or OTUs, are found solely in the sample of interest.

Skewness testing was carried out in Excel 2013 using the SKEW function which implements the Fisher–Pearsons test in order to determine whether the distribution of the data was skewed towards either end with the null hypothesis being normal symmetrical distribution. Significance of this was carried out by calculating the test statistic, where the test statistic is > 2 or < − 2 the skewness is considered significant, p-value = 0.05^80^.

Non-paired Student’s t-tests were carried out using the embedded function in R^81^ having first checked that the data showed the correct homoscedasticity using Fishers F-test, also in R.

Testing for correlation between data sets was carried out using Pearsons correlation coefficient testing via the embedded function in R.

## Supplementary Information

Supplementary Information.

## Data Availability

The datasets analysed during the current study are available in the NCBI sequence read archive as bioproject PRJNA775958 : Lincolnshire Limestone Metagenome, sample accession numbers are SAMN22636278 (St. Botolph-by_Bargate, damaged), SAMN22636279 (St. Botolph-by_Bargate, undamaged), SAMN22636280 (Burton Pedwardine church, damaged), SAMN22636281 (Burton Pedwardine church, undamaged), SAMN22636282 (Lincoln Cathedral, damaged), SAMN22636283 (Lincoln Cathedral, undamaged), SAMN22636284 (Saint Peter-at-Gowts, damaged) and SAMN22636285 (Saint Peter-at-Gowts, undamaged). All other data generated and analysed during the current study are available in this published article and its Supplementary Information files.
