# Supplementary material for: A metagenomic analysis of the bacterial microbiome of limestone, and the role of associated biofilms in the biodeterioration of heritage stone surfaces
Source: Sci Rep. 2022 Mar 22;12:4877. doi: 10.1038/s41598-022-08851-4 (PMC8940931; doi:10.1038/s41598-022-08851-4)
Supplement: Supplementary file 1 — Supplementary Information. [file 41598_2022_8851_MOESM1_ESM.pdf]

A metagenomic analysis of the bacterial microbiome of limestone, and the role of associated biofilms in the biodeterioration of heritage stone surfaces.

Philip J A Skipper[1*], Lynda K Skipper[1], Ronald A Dixon[2]
[1]School of History and Heritage, University of Lincoln, Lincoln, UK
[2]School of Life Sciences, University of Lincoln, Lincoln, UK
*** Correspondence:**
Corresponding Author
pskipper@lincoln.ac.uk

Supplementary Tables 1-4 and Supplementary Figure 1 are presented in this file.

Supplementary Table 1: Limestone microbiome species which were identified using the metagenomic data, including their % discovery across all samples and the surface(s) they were isolated from (damaged, undamaged or both).

species	% discovery across samples	Isolation surface	species	% discovery across samples	Isolation surface
Gemmiger prausnitzii	100	Both	*Corynebacterium pyruviciproducens*	37.5	Both
Propionibacterium granulosum	100	Both	*Ornithinibacter aureus*	37.5	Both
Acinetobacter lwoffii	100	Both	*Microbacterium humi*	37.5	Both
Collinsella aerofaciens	100	Both	*Kineococcus lusitanus*	37.5	Both
Micrococcus luteus	100	Both	*Corynebacterium striatum*	37.5	Both
Micrococcus terreus	100	Both	*Deinococcus reticulitermitis*	37.5	Both
Arthrobacter agilis	100	Both	*Rhizobium skierniewicense*	37.5	Both
Streptococcus thermophilus	100	Both	*Campylobacter ureolyticus*	37.5	Both
Finegoldia magna	100	Both	*Sphingomonas paucimobilis*	37.5	Both
Blautia glucerasea	100	Both	*Advenella mimigardefordensis*	37.5	Both
Actinophytocola sediminis	100	Both	*Streptococcus constellatus*	37.5	Undamaged
Pseudomonas fluorescens	87.5	Both	*Peptoniphilus grossensis*	37.5	Both
Flavobacterium lindanitolerans	87.5	Both	*Salinibacterium soli*	37.5	Both
Arthrobacter tumbae	87.5	Both	*Chryseobacterium nakagawai*	37.5	Both
Blastococcus jejuensis	87.5	Both	*Martelella radicis*	37.5	Both
Bifidobacterium pseudocatenulatum	87.5	Both	*Sphingomonas kyeonggiensis*	37.5	Both
Blautia wexlerae	87.5	Both	*Rhodococcus soli*	37.5	Both
Corynebacterium tuberculostearicum	87.5	Both	*Pantoea colletis*	37.5	Both
Streptococcus sanguinis	87.5	Both	*Ornithinimicrobium tianjinense*	37.5	Both
Arthrobacter subterraneus	87.5	Both	*Massilia eurypsychrophila*	37.5	Both
Propionibacterium acnes	87.5	Both	*Rhizobium binae*	37.5	Both
Peptococcus niger	87.5	Both	*Methylocapsa palsarum*	37.5	Both
Flavobacterium caeni	87.5	Both	*Streptococcus anginosus*	25	Both
Neisseria oralis	87.5	Both	*Cardiobacterium sp.Auto5*	25	Both
Ralstonia insidiosa	75	Both	*Spirosoma escalantus*	25	Damaged
Rhodobacter sphaeroides	75	Both	*Delftia acidovorans*	25	Both
Actinoplanes bacterium	75	Both	*Coprococcus catus*	25	Both
Actinobacillus bacterium	75	Both	*Blastococcus ginsenosidimutans*	25	Undamaged
Methylobacterium populi	75	Both	*Aerococcus sp.C216*	25	Both
Subdoligranulum bacterium	75	Both	*Lapillicoccus jejuensis*	25	Damaged
Flavobacterium sp.JSC-P2-223-10	75	Both	*Cronobacter sakazakii*	25	Both
Klebsiella sp.HGA0187	75	Both	*Bacillus muralis*	25	Both
Pseudomonas indica	75	Both	*Rhodobacter sp.BC14248*	25	Both
Dokdonella ginsengisoli	75	Both	*Streptococcus australis*	25	Damaged
Subtercola sp.Lor40	75	Both	*Bacteroides bacterium*	25	Both
Staphylococcus hominis	75	Both	*Terrabacter sp.2APm3*	25	Both
Blastococcus sp.13-106	75	Both	*Stenotrophomonas maltophilia*	25	Both
Afipia sp.42S5	75	Both	*Corynebacterium genitalium*	25	Damaged
Chryseobacterium sp.C15(2016)	75	Both	*Balneimonas flocculans*	25	Undamaged
Staphylococcus epidermidis	75	Both	*Pseudorhodoferax sp.KJ016*	25	Both
Anaerococcus nagyae	75	Both	*Bradyrhizobium sp.alfa7-PCA-E3-2*	25	Damaged
Kocuria palustris	75	Both	*Escherichia/Shigella flexneri*	25	Damaged
Gordonia polyisoprenivorans	75	Both	*Serratia sp.AMF2811*	25	Damaged
Pseudomonas aeruginosa	75	Both	*Negativicoccus sp.S5-A15*	25	Both
Okibacterium fritillariae	75	Both	*Lysobacter sp.YC6270*	25	Both
Brevundimonas bullata	75	Both	*Neisseria sp.2466a*	25	Damaged
Micrococcus sp.BAB-4450	75	Both	*Pontibacter sp.D14*	25	Both
Escherichia/Shigella sp.CCMICS	75	Both	*Hassallia sp.C76*	25	Damaged
Gemella morbillorum	75	Both	*Hansschlegelia sp.CHL1*	25	Damaged
Actinomycetospora sp.CB	75	Both	*Proteus penneri*	25	Damaged
Peptoniphilus sp.EL1	75	Both	*Lewinella sp.UA-AR0336*	25	Both
Microvirga soli	75	Both	*Haloactinopolyspora sp.Pao15*	25	Both
Lachnospiracea incertae sedis sp.AT12	75	Both	*Altererythrobacter sp.M0322*	25	Damaged
Bifidobacterium longum	75	Both	*Hyphomicrobium sp.ColF*	25	Undamaged
Ornithinicoccus hungaricus	75	Both	*Afipia carboxidovorans*	25	Damaged
Jannaschia rubra	75	Both	*Nocardioides marinisabuli*	25	Both
Blautia obeum	75	Both	*Bacillus pumilus*	25	Undamaged
Corynebacterium urealyticum	75	Both	*Kingella oralis*	25	Undamaged
Staphylococcus sp.16S	75	Both	*Citrobacter sp.B10(2014)*	25	Both
Streptococcus sp.3192A	62.5	Both	*Aeromicrobium panaciterrae*	25	Both
Pseudomonas tolaasii	62.5	Both	*Microbacterium pumilum*	25	Undamaged

species	% discovery across samples	Isolation surface	species	% discovery across samples	Isolation surface
Blautia obeum	62.5	Both	Flavisolibacter sp.ID1709	25	Both
Paracoccus sp.AP27	62.5	Both	Marisediminicola antarctica	25	Both
Nocardioides sp.9_67	62.5	Both	Sphingobacterium nematocida	25	Damaged
Acinetobacter calcoaceticus	62.5	Both	Prevotella bivia	25	Damaged
Actinomyces sp.ICM47	62.5	Both	Enterococcus faecalis	25	Damaged
Branchiibius sp.PSY066	62.5	Both	Phenylobacterium aquaticus	25	Undamaged
Phycicoccus sp.GP0608	62.5	Both	Roseateles sp.3HB-2p	25	Damaged
Undibacterium oligocarboniphilum	62.5	Both	Caldimonas taiwanensis	25	Both
Flavobacterium sp.CD13I-S2	62.5	Both	Salinibacterium sp.MJAU	25	Damaged
Adhaeribacter sp.120	62.5	Both	Brochothrix thermosphacta	25	Both
Propionibacterium namnetense	62.5	Both	Bosea sp.BRJL2	25	Both
Acinetobacter sp.25_SBR2	62.5	Both	Limnohabitans sp.Hippo3	25	Damaged
Kineosporia mesophila	62.5	Both	Staphylococcus bacterium	25	Both
Chryseobacterium arthrosphaerae	62.5	Both	Cellulomonas hominis	25	Damaged
Achromobacter xylosoxidans	62.5	Both	Derxia bacterium	25	Both
Roseateles depolymerans	62.5	Both	Bacillus cereus	25	Both
Sediminibacterium sp.HSD04	62.5	Both	Chryseobacterium taichungense	25	Both
Kineococcus bacterium	62.5	Both	Salinibacterium aurum	25	Both
Janibacter anophelis	62.5	Both	Clostridium XVIII ramosum	25	Both
Roseateles sp.K67	62.5	Both	Microbacterium natoriense	25	Both
Escherichia/Shigella coli	62.5	Both	Geodermatophilus siccatus	25	Damaged
Aurantimonas sp.HC-3	62.5	Both	Ornithinimicrobium sp.CMT700	25	Damaged
Anaerococcus octavius	62.5	Both	Gordonia paraffinivorans	25	Both
Neisseria mucosa	62.5	Both	Gordonia sp.NIHHS105	25	Both
Bradyrhizobium japonicum	62.5	Both	Facklamia hominis	25	Both
Arthrobacter flavus	62.5	Both	Lactobacillus curvatus	25	Both
Marinilactibacillus psychrotolerans	62.5	Both	Exiguobacterium sibiricum	25	Both
Friedmanniella capsulata	62.5	Both	Pseudomonas alcaligenes	25	Damaged
Brevundimonas sp.0312MAR21U9	62.5	Both	Rhizobium tumefaciens	25	Both
Solirubrobacter bacterium	62.5	Both	Pectobacterium carotovorum	25	Both
Staphylococcus haemolyticus	62.5	Both	Zhihengliuella flava	25	Both
Bifidobacterium adolescentis	62.5	Both	Micrococcus endophyticus	25	Both
Paraprevotella bacterium	62.5	Both	Dietzia sp.CSC19	25	Damaged
Varibaculum sp.Marseille-P2802	62.5	Both	Enterococcus durans	25	Damaged
Kocuria crystallopoietes	62.5	Both	Staphylococcus chromogenes	25	Both
Pedobacter panaciterrae	62.5	Both	Leifsonia sp.215	25	Damaged
Segetibacter koreensis	62.5	Both	Clavibacter sp.300	25	Damaged
Bifidobacterium pseudolongum	62.5	Both	Sporosarcina sp.305	25	Damaged
Humicoccus flavidus	62.5	Both	Haematobacter massiliensis	25	Both
Prevotella oris	62.5	Both	Sphingomonas sp.DUSK	25	Undamaged
Bifidobacterium bifidum	62.5	Both	Micrococcus yunnanensis	25	Damaged
Gemmiger formicilis	62.5	Both	Brevundimonas diminuta	25	Both
Derxia mirabilis	62.5	Both	Cellvibrio sp.MVW-40	25	Both
Marmoricola iriomotensis	62.5	Both	Proteus sp.SK3	25	Both
Actinomyces massiliensis	62.5	Both	Paracoccus carotinifaciens	25	Both
Blastococcus aggregatus	62.5	Both	Alkanindiges illinoisensis	25	Undamaged
Rhodopseudomonas robiniae	62.5	Both	Arthrobacter gandavensis	25	Both
Aciditerrimonas daechungensis	62.5	Both	Amaricoccus tamworthensis	25	Both
Ornithinicoccus hortensis	50	Both	Clostridium XlVa scindens	25	Both
Lysobacter bacterium	50	Both	Staphylococcus auricularis	25	Both
Curtobacterium sp.227-FB	50	Both	Nocardioides plantarum	25	Both
Pseudomonas libanensis	50	Both	Streptococcus agalactiae	25	Both
Corynebacterium durum	50	Both	Aquaspirillum arcticum	25	Both
Delftia tsuruhatensis	50	Both	Microbacterium pygmaeum	25	Both
Methylophilus bacterium	50	Both	Pseudonocardia hydrocarbonoxydans	25	Both
Erythrobacter bacterium	50	Both	Roseburia inulinivorans	25	Undamaged
Nocardioides rubriscoriae	50	Both	Arthrobacter monumenti	25	Both
Lactobacillus gasseri	50	Both	Deinococcus marmoris	25	Both
Pedobacter sp.2P1H2	50	Both	Sphingomonas mucosissima	25	Both
Eubacterium sp.ICM62	50	Both	Nocardioides hwasunensis	25	Both
Sorangium cellulosum	50	Both	Pelomonas aquatica	25	Undamaged
Bacillus simplex	50	Both	Quadrisphaera granulorum	25	Both
Lachnospiracea incertae sedis sp.canine	50	Both	Nocardioides lentus	25	Both
Granulicatella elegans	50	Both	Rothia terrae	25	Both

species	% discovery across samples	Isolation surface	species	% discovery across samples	Isolation surface
Dyadobacter sp.B2	50	Both	*Pedobacter insulae*	25	Undamaged
Dialister sp.S7MSR5	50	Both	*Nocardioides islandensis*	25	Both
Microlunatus sp.H22	50	Both	*Microbacterium laevaniformans*	25	Both
Massilia plicata	50	Both	*Rhodobacter sphaeroides*	25	Both
Clostridium sensu stricto perfringens	50	Both	*Kineococcus radiotolerans*	25	Undamaged
Friedmanniella sp.Pao16	50	Both	*Pseudomonas syringae*	25	Both
Abiotrophia defectiva	50	Both	*Lachnospiracea incertae sedis eligens*	25	Undamaged
Rothia sp.B18	50	Both	*Rothia mucilaginosa*	25	Undamaged
Pseudomonas poae	50	Both	*Polaromonas sp.JS666*	25	Both
Aurantimonas sp.4M3-2	50	Both	*Calothrix sp.PCC*	25	Undamaged
Friedmanniella aerolata	50	Both	*Patulibacter rosea*	25	Undamaged
Actinotalea sp.734H3	50	Both	*Neisseria elongata*	25	Both
Ralstonia pickettii	50	Both	*Pseudonocardia seranimata*	25	Undamaged
Kocuria kristinae	50	Both	*Janibacter badiiscoriae*	25	Both
Streptococcus gastrococcus	50	Both	*Chryseobacterium yonginense*	25	Both
Sporichthya polymorpha	50	Both	*Novosphingobium dongtanensis*	25	Both
Acinetobacter haemolyticus	50	Both	*Saxeibacter panacisegetis*	25	Both
Arthrobacter cumminsii	50	Both	*Blautia faecis*	25	Undamaged
Mesorhizobium mediterraneum	50	Both	*Gulosibacter chungangensis*	25	Undamaged
Rhodococcus sp.320	50	Damaged	*Sphingomonas cynarae*	25	Both
Phyllobacterium sp.JCM	50	Both	*Ralstonia psychrotolerans*	25	Both
Modestobacter lapidis	50	Both	*Dongia soli*	25	Both
Saxeibacter bacterium	50	Both	*Mycobacterium arabiense*	25	Both
Anaerosphaera bacterium	50	Both	*Friedmanniella lucida*	25	Damaged
Dietzia cinnamea	50	Both	*Porphyromonas catoniae*	25	Both
Brevundimonas faecalis	50	Both	*Acidipila rosea*	25	Both
Clostridium XlVa xylanolyticus	50	Both	*Branchiibius cervicis*	25	Both
Veillonella atypica	50	Both	*Dialister invisus*	25	Both
Corynebacterium suicordis	50	Both	*Sphingobacterium faecium*	25	Both
Stenotrophomonas terrae	50	Both	*Dolosigranulum pigrum*	25	Undamaged
Aurantimonas ureilytica	50	Both	*Sphingomonas astaxanthinifaciens*	25	Both
Coprococcus comes	50	Both	*Parvimonas micra*	25	Both
Microbacterium kribbense	50	Both	*Clostridium XVIII spiroforme*	25	Both
Roseomonas frigidaquae	50	Both	*Rhodoplanes piscinae*	25	Both
Porphyromonas bennonis	50	Both	*Anaerococcus murdochii*	25	Both
Kocuria rhizophila	50	Both	*Haemophilus parainfluenzae*	25	Undamaged
Proteus mirabilis	50	Both	*Arenimonas oryziterrae*	25	Both
Bifidobacterium breve	50	Both	*Terrabacter aeriphilus*	25	Both
Blastococcus saxobsidens	50	Both	*Arthrobacter halodurans*	25	Both
Tepidimicrobium coagulans	50	Both	*Deinococcus aerolatus*	25	Both
Moraxella nonliquefaciens	50	Both	*Arthrobacter aestuarii*	25	Both
Friedmanniella okinawensis	50	Both	*Dietzia papillomatosis*	25	Both
Psychrobacter immobilis	50	Both	*Negativicoccus succinicivorans*	25	Both
Kocuria phoenicis	50	Both	*Nocardioides caricicola*	25	Damaged
Rhodococcus cerastii	50	Both	*Porphyromonas asaccharolytica*	25	Both
Methylobacterium bullatum	50	Both	*Sphingomonas rubra*	25	Undamaged
Dyadobacter sediminis	50	Both	*Corynebacterium pilbarense*	25	Both
Burkholderia metalliresistens	50	Both	*Marmoricola korecus*	25	Undamaged
Bacillus licheniformis	50	Damaged	*Hymenobacter psychrophilus*	25	Both
Neisseria perflava	37.5	Both	*Hymenobacter elongatus*	25	Both
Corynebacterium glucuronolyticum	37.5	Undamaged	*Peptoniphilus coxii*	25	Both
Staphylococcus caprae	37.5	Both	*Saxeibacter lacteus*	25	Undamaged
Microbacterium lacticum	37.5	Both	*Sphingomonas roseiflava*	25	Undamaged
Kocuria bacterium	37.5	Both	*Streptococcus dentisani*	25	Both
Pseudonocardia zijingensis	37.5	Both	*Roseburia intestinalis*	25	Both
Kineococcus sp.psc3	37.5	Both	*Hymenobacter yonginensis*	25	Both
Paenibacillus sp.1105	37.5	Damaged	*Anaerococcus senegalensis*	25	Both
Ralstonia proteobacterium	37.5	Both	*Propionibacterium avidum*	25	Both
Prevotella salivae	37.5	Both	*Salmonella enterica*	25	Undamaged
Lachnospiracea incertae sedis Dorea	37.5	Both	*Neisseria cinerea*	25	Damaged
Humicoccus bacterium	37.5	Both	*Frondihabitans sucicola*	25	Undamaged
Roseburia sp.831b	37.5	Both	*Massilia kyonggiensis*	25	Both
Hymenobacter perfusus	37.5	Both	*Hymenobacter qilianensis*	25	Both
Arthrobacter arilaitensis	37.5	Both	*Demetria marinus*	25	Undamaged

species	% discovery across samples	Isolation surface	species	% discovery across samples	Isolation surface
Pseudomonas argentinensis	37.5	Damaged	*Hymenobacter tibetensis*	25	Undamaged
Nocardioides jensenii	37.5	Both	*Chryseobacterium arachidis*	25	Both
Corynebacterium tuscaniense	37.5	Both	*Flavobacterium plurextorum*	25	Undamaged
Actinoplanes digitatis	37.5	Both	*Hymenobacter arcticus*	25	Both
Mucilaginibacter sp.*G03*	37.5	Both	*Hymenobacter kanuolensis*	25	Undamaged
Quadrisphaera bacterium	37.5	Both	*Paenalcaligenes suwonensis*	25	Both
Amaricoccus sp.*YIM125*	37.5	Both	*Pedobacter jejuensis*	25	Both
Haemophilus influenzae	37.5	Damaged	*Pedobacter glacialis*	25	Both
Bosea sp.*CC11C2*	37.5	Both	*Ralstonia syzygii*	25	Undamaged
Mesorhizobium sp.*ADC-19B*	37.5	Both	*Chelatococcus caeni*	25	Both
Aurantimonas glaciistagni	37.5	Both	*Clavibacter michiganensis*	25	Undamaged
Sphingomonas cucumeris	37.5	Both	*Frigoribacterium endophyticum*	25	Undamaged
Rheinheimera tangshanensis	37.5	Both	*Lysobacter mobilis*	25	Undamaged
Methylibium petroleiphilum	37.5	Both	*Roseomonas tokyonensis*	25	Undamaged
Roseateles chitosanitabida	37.5	Both	*Porphyromonas pasteri*	25	Undamaged
Comamonas sp.*IDO2*	37.5	Both	*Sphingomonas zeae*	25	Damaged
Rathayibacter sp.*JSM*	37.5	Both	*Nocardioides antarcticus*	25	Undamaged
Modestobacter sp.*I12A-02988*	37.5	Both	*Sphingomonas psychrolutea*	25	Both
Gordonibacter sp.*S475*	37.5	Damaged	*Mesorhizobium cantuariense*	25	Both
Nesterenkonia sp.*CPA-95*	37.5	Both	*Escherichia/Shigella agona*	25	Both
Kitasatospora sp.*1C-32*	37.5	Both	*Psychrobacillus psychrodurans*	25	Damaged
Neisseria cerebrosus	37.5	Both	*Curtobacterium flaccumfaciens*	25	Both
Stenotrophomonas sp.*9Kp10a*	37.5	Both	*Metascardovia* sp.*OB7196*	12.5	Damaged
Delftia sp.*ALBL_009*	37.5	Both	*Rubrivivax* sp.*YIT*	12.5	Damaged
Acidovorax sp.*ALBL_202*	37.5	Both	*Virgisporangium ochraceum*	12.5	Undamaged
Chryseobacterium indoltheticum	37.5	Both	*Devosia submarina*	12.5	Undamaged
Leifsonia sp.*2EM3*	37.5	Both	*Lachnospiracea incertae sedis hydrogenotrophica*	12.5	Damaged
Chryseobacterium joostei	37.5	Both	*Clostridium sensu stricto celatum*	12.5	Damaged
Sphingomonas canadensis	37.5	Both	*Duganella* sp.*MsC-12-4CB4-02*	12.5	Damaged
Microbacterium trichothecenolyticum	37.5	Both	*Piscicoccus crocodyli*	12.5	Damaged
Pedobacter suwonensis	37.5	Both	*Aestuariimicrobium thiooxidans*	12.5	Damaged
Pantoea septica	37.5	Damaged	*Smithella* sp.*16S*	12.5	Undamaged
Bacteroides fragilis	37.5	Both	*Nitrospira* sp.*clone*	12.5	Damaged
Prevotella timonensis	37.5	Both	*Escherichia/Shigella boydii*	12.5	Damaged
Sphingomonas koreensis	37.5	Both	*Serratia aquatilis*	12.5	Damaged
Pantoea agglomerans	37.5	Both	*Pseudomonas synxantha*	12.5	Damaged
Bacillus toyonensis	37.5	Both	*Halococcus morrhuae*	12.5	Damaged
Enterobacter cloacae	37.5	Both	*Serratia* sp.*NJ-71*	12.5	Damaged
Sphingobacterium sp.*FM2*	37.5	Both	*Bacillus longiquaesitum*	12.5	Damaged
Pseudomonas stutzeri	37.5	Both	*Kribbella sancallistae*	12.5	Undamaged
Psychrobacter sp.*44(2016)*	37.5	Both	*Nocardioides hungaricus*	12.5	Undamaged
Serratia bacterium	37.5	Both	*Nitrosospira briensis*	12.5	Damaged
Plantibacter sp.*H53*	37.5	Both	*Anaerorhabdus Hepatoplasma*	12.5	Damaged
Actinoplanes lichenis	37.5	Both	*Corynebacterium appendicis*	12.5	Damaged
Haemophilus parahaemolyticus	37.5	Both	*Streptomyces lazureus*	12.5	Damaged
Derxia sp.*CB*	37.5	Both	*Anaplasma Wolbachia*	12.5	Undamaged
Acidiphilium sp.*N29*	37.5	Both	*Sphingomonas trueperi*	12.5	Damaged
Friedmanniella lacustris	37.5	Both	*Kineococcus bacterium*	12.5	Damaged
Arthrobacter roseus	37.5	Both	*Methylobacterium* sp.*14-324*	12.5	Damaged
Lactobacillus iners	37.5	Both	*Hymenobacter bacterium*	12.5	Damaged
Hymenobacter soli	37.5	Both	*Arthrobacter cumminsii*	12.5	Damaged
Roseomonas aquatica	37.5	Both	*Brevibacterium paucivorans*	12.5	Damaged
Chryseobacterium hominis	37.5	Both	*Rhodobacter* sp.*D4028*	12.5	Damaged
Brevundimonas terrae	37.5	Both	*Natroniella rupestris*	12.5	Damaged
Piscinibacter aquaticus	37.5	Both	*Legionella* sp.*legS052*	12.5	Damaged
Chryseobacterium haifense	37.5	Both	*Jejuia arisari*	12.5	Damaged
Streptococcus parasanguinis	37.5	Undamaged	*Leptothrix* sp.*OTSz_A_252*	12.5	Damaged
Sphingomonas wittichii	37.5	Both	*Mesorhizobium* sp.*OTSz_M_287*	12.5	Damaged
Exiguobacterium sp.*AT1b*	37.5	Both	*Schlegelella aquatica*	12.5	Damaged
Solirubrobacter ginsenosidimutans	37.5	Undamaged	*Advenella kashmirensis*	12.5	Undamaged
Methylobacterium gossipiicola	37.5	Both	*Staphylococcus xylosus*	12.5	Damaged
Blastococcus endophyticus	37.5	Both	*Micrococcus halobius*	12.5	Undamaged
Friedmanniella flava	37.5	Both	*Altererythrobacter* sp.*CTDB1*	12.5	Damaged
Pseudonocardia antitumoralis	37.5	Both	*Ilumatobacter* sp.*T2-YC6790*	12.5	Damaged

species	% discovery across samples	Isolation surface	species	% discovery across samples	Isolation surface
Friedmanniella sagamiharensis	37.5	Damaged	*Gemmata sp.Br1-2*	12.5	Undamaged
Actinomycetospora cinnamomea	37.5	Both	*Aeromicrobium sp.CNRD02*	12.5	Damaged
Piscicoccus intestinalis	37.5	Both	*Brevinema flagrans*	12.5	Damaged
Paracoccus kocurii	37.5	Both	*Massilia sp.sptzw26*	12.5	Damaged
Sporosarcina luteola	37.5	Both	*Veillonella dispar*	12.5	Damaged
Psychrobacter adeliensis	37.5	Both	*Veillonella parvula*	12.5	Undamaged
Hansschlegelia plantiphila	37.5	Both	*Fusobacterium nucleatum*	12.5	Damaged
Thalassobius gelatinovorus	37.5	Both	*Eubacterium brachy*	12.5	Undamaged
Hymenobacter algoricola	37.5	Both	*Streptococcus cristatus*	12.5	Damaged
Cardiobacterium hominis	12.5	Damaged	*Ruminococcus callidus*	12.5	Damaged
Streptococcus mitis	12.5	Damaged	*Lachnospiracea incertae sedis torques*	12.5	Undamaged
Leptotrichia sp.PTE15	12.5	Damaged	*Bifidobacterium angulatum*	12.5	Undamaged
Aggregatibacter segnis	12.5	Undamaged	*Couchioplanes caeruleus*	12.5	Damaged
Rhodoplanes sp.JA527	12.5	Damaged	*Pseudomonas azotoformans*	12.5	Undamaged
Acinetobacter parvus	12.5	Damaged	*Bifidobacterium gallicum*	12.5	Damaged
Caenimonas sp.TSX9-5	12.5	Damaged	*Carnobacterium mobile*	12.5	Undamaged
Providencia sp.DF1SB	12.5	Damaged	*Planifilum fimeticola*	12.5	Damaged
Solibacillus PM-38	12.5	Damaged	*Microlunatus ginsengisoli*	12.5	Undamaged
Propionibacterium sp.434-HC2	12.5	Damaged	*Loktanella atrilutea*	12.5	Damaged
Turicibacter sanguinis	12.5	Damaged	*Parabacteroides johnsonii*	12.5	Undamaged
Micrococcus flavus	12.5	Damaged	*Agrococcus citreus*	12.5	Damaged
Granulicatella para-adiacens	12.5	Damaged	*Microbacterium lacus*	12.5	Undamaged
Sphingomonas sp.14	12.5	Damaged	*Glaciibacter superstes*	12.5	Undamaged
Anaerovorax bacterium	12.5	Damaged	*Sphingomonas japonica*	12.5	Undamaged
Enterococcus viikkiensis	12.5	Undamaged	*Cellvibrio mixtus*	12.5	Undamaged
Rhizobacter sp.CR2	12.5	Damaged	*Acidovorax anthurii*	12.5	Damaged
Bradyrhizobium liaoningense	12.5	Damaged	*Arthrobacter phytoseiuli*	12.5	Undamaged
Sphingomonas humi	12.5	Damaged	*Providencia vermicola*	12.5	Damaged
Pantoea ananatis	12.5	Damaged	*Devosia subaequoris*	12.5	Undamaged
Haemophilus haemolyticus	12.5	Damaged	*Agrococcus jejuensis*	12.5	Undamaged
Ornithinimicrobium pekingense	12.5	Damaged	*Actinomycetospora chiangmaiensis*	12.5	Damaged
Nocardioides aquiterrae	12.5	Damaged	*Rhodobacter maris*	12.5	Damaged
Devosia sp.Axs16	12.5	Undamaged	*Chryseobacterium gregarium*	12.5	Undamaged
Morganella morganii	12.5	Undamaged	*Kribbella catacumbae*	12.5	Undamaged
Arthrobacter bacterium	12.5	Damaged	*Deinococcus aquatilis*	12.5	Damaged
Roseococcus sp.JNUS-15	12.5	Damaged	*Roseburia faecis*	12.5	Undamaged
Turicibacter sp.HGA0205	12.5	Damaged	*Cellulomonas denverensis*	12.5	Damaged
Prevotella sp.HGA0217	12.5	Damaged	*Ornithinimicrobium kibberense*	12.5	Undamaged
Devosia glacialis	12.5	Damaged	*Mycobacterium aubagnense*	12.5	Undamaged
Quadrisphaera sp.THG-DM1	12.5	Undamaged	*Nocardioides dubius*	12.5	Damaged
Bradyrhizobium elkanii	12.5	Damaged	*Brevundimonas kwangchunensis*	12.5	Undamaged
Anaerococcus hydrogenalis	12.5	Damaged	*Leifsonia naganoensis*	12.5	Undamaged
Kytococcus sedentarius	12.5	Undamaged	*Nocardioides furvisabuli*	12.5	Undamaged
Thermomonas brevis	12.5	Undamaged	*Methylobacterium jeotgali*	12.5	Undamaged
Luteimonas terricola	12.5	Damaged	*Salinibacterium xinjiangense*	12.5	Damaged
Acidocella facilis	12.5	Damaged	*Skermanella aerolata*	12.5	Damaged
Pseudomonas proteobacterium	12.5	Undamaged	*Nocardioides insulae*	12.5	Damaged
Mycobacterium phocaicum	12.5	Undamaged	*Pseudonocardia endophytica*	12.5	Undamaged
Paenibacillus darwinianus	12.5	Damaged	*Methylobacterium iners*	12.5	Undamaged
Craurococcus sp.HM28-1	12.5	Damaged	*Corynebacterium massiliense*	12.5	Damaged
Bacillus aerophilus	12.5	Damaged	*Marmoricola bigeumensis*	12.5	Damaged
Rhizobacter sp.7B-213	12.5	Undamaged	*Nocardioides dilutus*	12.5	Damaged
Leifsonia bacterium	12.5	Damaged	*Knoellia aerolata*	12.5	Damaged
Roseibacterium sp.HME9693	12.5	Damaged	*Nocardioides fonticola*	12.5	Undamaged
Amorphus sp.JL1095	12.5	Damaged	*Deinococcus radiomollis*	12.5	Damaged
Pelomonas puraquae	12.5	Damaged	*Deinococcus claudionis*	12.5	Damaged
Klebsiella pneumoniae	12.5	Damaged	*Massilia niastensis*	12.5	Damaged
Peptostreptococcus stomatis	12.5	Damaged	*Brevibacterium aurantiacum*	12.5	Undamaged
Rothia dentocariosa	12.5	Undamaged	*Desemzia incerta*	12.5	Damaged
Wautersiella sp.SCU-B169	12.5	Undamaged	*Legionella worsleiensis*	12.5	Damaged
Beijerinckia sp.Pao66	12.5	Damaged	*Aeromicrobium fastidiosum*	12.5	Damaged
Actinotalea fermentans	12.5	Damaged	*Methylobacterium extorquens*	12.5	Undamaged
Bradyrhizobium palustris	12.5	Damaged	*Rhizobium fabrum*	12.5	Undamaged
Alistipes inops	12.5	Damaged	*Terriglobus saanensis*	12.5	Undamaged

species	% discovery across samples	Isolation surface	species	% discovery across samples	Isolation surface
Arthrobacter echini	12.5	Damaged	*Erythrobacter litoralis*	12.5	Undamaged
Herbaspirillum sp.*ES2-54*	12.5	Damaged	*Kribbella flavida*	12.5	Damaged
Pantoea sp.*136A*	12.5	Damaged	*Intrasporangium calvum*	12.5	Damaged
Nitrobacter sp.*NKU*	12.5	Damaged	*Alicycliphilus denitrificans*	12.5	Damaged
Acidovorax bacterium	12.5	Damaged	*Brachybacterium faecium*	12.5	Undamaged
Bordetella sp.*UASWS0941*	12.5	Damaged	*Sanguibacter keddieii*	12.5	Undamaged
Pusillimonas sp.*UASWS0960*	12.5	Damaged	*Arthrobacter phenanthrenivorans*	12.5	Undamaged
Enterococcus faecium	12.5	Damaged	*Roseburia hominis*	12.5	Damaged
Vogesella perlucida	12.5	Damaged	*Actinobacillus succinogenes*	12.5	Undamaged
Patulibacter sp.*R16*	12.5	Undamaged	*Escherichia/Shigella dysenteriae*	12.5	Undamaged
Pedobacter luteus	12.5	Damaged	*Selenomonas sputigena*	12.5	Undamaged
Curvibacter fontanus	12.5	Damaged	*Psychrobacter cryohalolentis*	12.5	Damaged
Porphyrobacter mercurialis	12.5	Damaged	*Pseudomonas entomophila*	12.5	Undamaged
Aquamicrobium populi	12.5	Damaged	*Prevotella melaninogenica*	12.5	Undamaged
Coenonia sp.*LS-2015b*	12.5	Damaged	*Pantoea vagans*	12.5	Undamaged
Salinarimonas sp.*M016010*	12.5	Damaged	*Salmonella oxytoca*	12.5	Undamaged
Mycobacterium litorale	12.5	Damaged	*Streptococcus oligofermentans*	12.5	Damaged
Marmoricola sp.*BN130122*	12.5	Undamaged	*Aeromicrobium halocynthiae*	12.5	Undamaged
Williamsia sp.*ARP1*	12.5	Damaged	*Enterococcus gallinarum*	12.5	Undamaged
Mycobacterium sp.*27486-12*	12.5	Damaged	*Blautia hansenii*	12.5	Damaged
Luteipulveratus sp.*M20-45*	12.5	Damaged	*Pseudonocardia spinosa*	12.5	Damaged
Janibacter cremeus	12.5	Damaged	*Aerococcus viridans*	12.5	Undamaged
Chryseobacterium hispalense	12.5	Damaged	*Pseudonocardia alaniniphila*	12.5	Damaged
Serinibacter sp.*K3-2*	12.5	Damaged	*Actinomyces oris*	12.5	Undamaged
Hoeflea sp.*JSM*	12.5	Undamaged	*Providencia sneebia*	12.5	Undamaged
Fodinicola sp.*I14A-00812*	12.5	Damaged	*Novosphingobium troitsensis*	12.5	Damaged
Agreia sp.*I12A-02597*	12.5	Damaged	*Lysobacter korlensis*	12.5	Undamaged
Rummeliibacillus sp.*I13B-01798*	12.5	Damaged	*Methylobacterium phyllostachyos*	12.5	Damaged
Trueperella sp.*S350*	12.5	Damaged	*Pseudonocardia xishanensis*	12.5	Undamaged
Parabacteroides sp.*S449*	12.5	Damaged	*Leifsonia moechotypicola*	12.5	Undamaged
Adlercreutzia sp.*S45*	12.5	Damaged	*Williamsia phyllosphaerae*	12.5	Damaged
Enterobacter adecarboxylata	12.5	Damaged	*Ornithinimicrobium murale*	12.5	Damaged
Bacillus thermoamylovorans	12.5	Damaged	*Nocardioides ginsengisegetis*	12.5	Damaged
Parabacteroides distasonis	12.5	Damaged	*Nocardioides ginsengagri*	12.5	Undamaged
Amycolatopsis marina	12.5	Damaged	*Agrococcus carbonis*	12.5	Damaged
Enterobacter xiangfangensis	12.5	Damaged	*Acidovorax terrae*	12.5	Undamaged
Gillisia sp.*M5A-3*	12.5	Damaged	*Pseudorhodoferax aquiterrae*	12.5	Damaged
Epilithonimonas sp.*PDD-58b-23*	12.5	Damaged	*Rhodobacter halotolerans*	12.5	Undamaged
Pseudomonas rhizosphaerae	12.5	Damaged	*Leuconostoc pseudomesenteroides*	12.5	Damaged
Frondihabitans sp.*PDD-63b-8*	12.5	Damaged	*Micromonospora abujensis*	12.5	Undamaged
Frigoribacterium sp.*PDD-69b-5*	12.5	Damaged	*Catellibacterium lanyuensis*	12.5	Undamaged
Variovorax sp.*PDD-69b-8*	12.5	Damaged	*Salinibacterium soli*	12.5	Undamaged
Thermoactinomyces sp.*T36*	12.5	Damaged	*Dyadobacter arcticus*	12.5	Undamaged
Trichococcus pasteurii	12.5	Damaged	*Hymenobacter saemangeumensis*	12.5	Undamaged
Serinicoccus sp.*CR-19*	12.5	Damaged	*Paracoccus rhizosphaerae*	12.5	Damaged
Ureibacillus thermosphaericus	12.5	Damaged	*Algoriphagus sdotyamensis*	12.5	Undamaged
Acidovorax delafieldii	12.5	Undamaged	*Tepidimonas fonticaldi*	12.5	Undamaged
Ignatzschineria sp.*077229*	12.5	Damaged	*Luteolibacter yonseiensis*	12.5	Undamaged
Planococcaceae incertae sedis boronitolerans	12.5	Damaged	*Aurantimonas jatrophae*	12.5	Undamaged
Bacillus oleronius	12.5	Damaged	*Leifsonia aerilata*	12.5	Undamaged
Streptomyces flavogriseus	12.5	Damaged	*Roseomonas aerophila*	12.5	Undamaged
Stenotrophomonas humi	12.5	Damaged	*Geodermatophilus taihuensis*	12.5	Undamaged
Porphyrobacter sp.*LB1-11*	12.5	Damaged	*Nocardioides salsibiostraticola*	12.5	Undamaged
Rhizobium sp.*Mol*	12.5	Damaged	*Lysobacter ginsengisoli*	12.5	Damaged
Rhizobium giardinii	12.5	Damaged	*Actinaurispora siamensis*	12.5	Undamaged
Corynebacterium aurimucosum	12.5	Undamaged	*Actinomycetospora chlora*	12.5	Undamaged
Corynebacterium imitans	12.5	Damaged	*Porphyromonas uenonis*	12.5	Undamaged
Pseudoxanthomonas sp.*ALBL_054*	12.5	Undamaged	*Prevotella buccalis*	12.5	Undamaged
Rhodopseudomonas sp.*BRJL9*	12.5	Damaged	*Austwickia chelonae*	12.5	Damaged
Arthrobacter bacterium	12.5	Damaged	*Bifidobacterium biavatii*	12.5	Damaged
Phycicoccus ochangensis	12.5	Damaged	*Actinomycetospora iriomotensis*	12.5	Undamaged
Erwinia cedenensis	12.5	Damaged	*Cellulomonas soli*	12.5	Damaged
Ramlibacter sp.*YS*	12.5	Damaged	*Dialister micraerophilus*	12.5	Undamaged
Ornithinimicrobium humiphilum	12.5	Damaged	*Enhydrobacter osloensis*	12.5	Undamaged

species	% discovery across samples	Isolation surface	species	% discovery across samples	Isolation surface
Massilia sp.2PM3lan	12.5	Damaged	*Brachybacterium paraconglomeratum*	12.5	Damaged
Sphingobium limneticum	12.5	Damaged	*Capnocytophaga sputigena*	12.5	Damaged
Chryseobacterium UYP8	12.5	Damaged	*Brevundimonas vesicularis*	12.5	Damaged
Kocuria carniphila	12.5	Damaged	*Simplicispira psychrophila*	12.5	Undamaged
Rhodococcus fascians	12.5	Undamaged	*Sphingobacterium mizutaii*	12.5	Damaged
Acinetobacter indicus	12.5	Undamaged	*Pedobacter piscium*	12.5	Undamaged
Paracoccus yeei	12.5	Undamaged	*Sphingomonas adhaesiva*	12.5	Undamaged
Pseudomonas taiwanensis	12.5	Damaged	*Sphingomonas echinoides*	12.5	Damaged
Corynebacterium flavescens	12.5	Damaged	*Paracoccus seriniphilus*	12.5	Damaged
Chryseobacterium treverense	12.5	Damaged	*Sphingomonas jaspsi*	12.5	Undamaged
Microbacterium suwonense	12.5	Damaged	*Rhizobium daejeonense*	12.5	Damaged
Leifsonia lichenia	12.5	Damaged	*Sphingobium amiense*	12.5	Undamaged
Brevibacterium sp.AIC-5	12.5	Damaged	*Oxalicibacterium solurbis*	12.5	Undamaged
Curvibacter sp.S201	12.5	Damaged	*Bradyrhizobium boonkerdii*	12.5	Undamaged
Lactobacillus coryniformis	12.5	Damaged	*Acidovorax konjaci*	12.5	Undamaged
Thermofilum archaeon	12.5	Damaged	*Enterococcus hirae*	12.5	Undamaged
Lactobacillus agilis	12.5	Damaged	*Microbacterium maritypicum*	12.5	Undamaged
Leuconostoc mesenteroides	12.5	Damaged	*Acinetobacter baylyi*	12.5	Both
Erwinia rhapontici	12.5	Undamaged	*Flavobacterium subsaxonicum*	12.5	Undamaged
Oerskovia sp.CON39-30	12.5	Damaged	*Phenylobacterium mobile*	12.5	Undamaged
Agrococcus baldri	12.5	Damaged	*Solobacterium moorei*	12.5	Undamaged
Streptococcus pasteurianus	12.5	Undamaged	*Alkanindiges hongkongensis*	12.5	Damaged
Delftia lacustris	12.5	Damaged	*Actinoplanes auranticolor*	12.5	Damaged
Yimella sp.py1292	12.5	Damaged	*Lysobacter dokdonensis*	12.5	Undamaged
Curvibacter sp.DCY110	12.5	Damaged	*Solibacillus isronensis*	12.5	Damaged
Salinispora sp.NHF45	12.5	Undamaged	*Rhizobium soli*	12.5	Damaged
Macrochaete lichenoides	12.5	Damaged	*Labrys wisconsinensis*	12.5	Undamaged
Dermacoccus sp.CMT48	12.5	Damaged	*Pseudonocardia saturnea*	12.5	Undamaged
Aquamicrobium sp.342B3_12ECASO	12.5	Damaged	*Phyllobacterium aestuarii*	12.5	Damaged
Rickettsia Rickettsia	12.5	Damaged	*Jannaschia seohaensis*	12.5	Damaged
Corynebacterium variabile	12.5	Damaged	*Streptococcus gordonii*	12.5	Undamaged
Enterobacter asburiae	12.5	Damaged	*Modestobacter marinus*	12.5	Damaged
Pseudomonas putida	12.5	Undamaged	*Brevibacterium pityocampae*	12.5	Damaged
Pseudomonas fulva	12.5	Damaged	*Serinicoccus profundi*	12.5	Damaged
Clostridium sensu stricto novyi	12.5	Undamaged	*Rhizobium rosettiformans*	12.5	Undamaged
Streptococcus tigurinus	12.5	Damaged	*Microbacterium aurantiacum*	12.5	Damaged
Atopobium parvulum	12.5	Damaged	*Pseudonocardia autotrophica*	12.5	Damaged
Cosenzaea myxofaciens	12.5	Damaged	*Sphingomonas hankookensis*	12.5	Damaged
Actinokineospora sp.R434	12.5	Damaged	*Zhihengliuella salsuginis*	12.5	Damaged
Rathayibacter tanaceti	12.5	Damaged	*Sphingomonas hunanensis*	12.5	Undamaged
Pedobacter duraquae	12.5	Damaged	*Eubacterium umeaense*	12.5	Damaged
Labedella gwakjiensis	12.5	Damaged	*Pseudonocardia adelaidensis*	12.5	Damaged
Acidovorax avenae	12.5	Damaged	*Vibrio casei*	12.5	Damaged
Lactobacillus crispatus	12.5	Damaged	*Naxibacter suwonensis*	12.5	Undamaged
Pantoea sp.MDMC194	12.5	Damaged	*Methylobacterium cerastii*	12.5	Damaged
Chryseobacterium soldanellicola	12.5	Damaged	*Anaerostipes hadrus*	12.5	Undamaged
Cronobacter malonaticus	12.5	Damaged	*Hymenobacter glaciei*	12.5	Damaged
Cedecea davisae	12.5	Damaged	*Bacillus frigoritolerans*	12.5	Damaged
Pseudochrobactrum asaccharolyticum	12.5	Damaged	*Peptoniphilus tyrrelliae*	12.5	Undamaged
Bradyrhizobium faecalis	12.5	Damaged	*Acinetobacter tandoii*	12.5	Undamaged
Pseudacidovorax intermedius	12.5	Damaged	*Pseudomonas viridiflava*	12.5	Damaged
Roseateles toxinivorans	12.5	Damaged	*Sphingomonas ginsenosidivorax*	12.5	Undamaged
Variovorax ginsengisoli	12.5	Damaged	*Fusobacterium periodonticum*	12.5	Damaged
Geobacillus caldoxylosilyticus	12.5	Damaged	*Microvirga lotononidis*	12.5	Damaged
Exiguobacterium aurantiacum	12.5	Undamaged	*Rhizobium tarimense*	12.5	Undamaged
Rhodococcus coeliaca	12.5	Damaged	*Isoptericola nanjingensis*	12.5	Damaged
Chryseobacterium M8	12.5	Damaged	*Psychrobacter pulmonis*	12.5	Undamaged
Rhizobium galegae	12.5	Damaged	*Arenimonas metalli*	12.5	Undamaged
Anaplasma pipientis	12.5	Undamaged	*Cellulomonas ferrariae*	12.5	Damaged
Bacillus kokeshiiformis	12.5	Damaged	*Planococcus halocryophilus*	12.5	Damaged
Staphylococcus vitulinus	12.5	Damaged	*Massilia namucuonensis*	12.5	Damaged
Mycobacterium fortuitum	12.5	Damaged	*Frondihabitans suwonensis*	12.5	Damaged
Psychrobacter nivimaris	12.5	Damaged	*Sphingobium czechense*	12.5	Undamaged
Kocuria salsicia	12.5	Damaged	*Peptoniphilus timonensis*	12.5	Damaged

species	% discovery across samples	Isolation surface	species	% discovery across samples	Isolation surface
Enhydrobacter sp.Td-10	12.5	Damaged	*Novosphingobium lindaniclasticum*	12.5	Undamaged
Bradyrhizobium daqingense	12.5	Damaged	*Anaerococcus obesiensis*	12.5	Damaged
Staphylococcus kloosii	12.5	Damaged	*Aurantimonas phyllosphaerae*	12.5	Undamaged
Lactobacillus helveticus	12.5	Damaged	*Phaeobacter gallaeciensis*	12.5	Undamaged
Erwinia coffeiphila	12.5	Undamaged	*Rathayibacter iranicus*	12.5	Undamaged
Clostridium sensu stricto beijerinckii	12.5	Damaged	*Mucilaginibacter calamicampi*	12.5	Damaged
Leuconostoc citreum	12.5	Damaged	*Gordonia terrae*	12.5	Undamaged
Providencia rettgeri	12.5	Damaged	*Bordetella trematum*	12.5	Undamaged
Actinomyces johnsonii	12.5	Damaged	*Ralstonia solanacearum*	12.5	Damaged
Providencia stuartii	12.5	Damaged	*Sulfuricella denitrificans*	12.5	Undamaged
Serratia sp.F3-1-11	12.5	Damaged	*Micromonospora friuliensis*	12.5	Undamaged
Ralstonia sp.BAB-4439	12.5	Damaged	*Stenotrophomonas rhizophila*	12.5	Damaged
Escherichia/Shigella sp.BAB-5849	12.5	Damaged	*Corynebacterium casei*	12.5	Damaged
Staphylococcus saprophyticus	12.5	Damaged	*Mycobacterium insubricum*	12.5	Undamaged
Pantoea sp.S2	12.5	Damaged	*Agaricicola taiwanensis*	12.5	Undamaged
Virgibacillus sp.SP-2.7	12.5	Damaged	*Lactobacillus hominis*	12.5	Damaged
Enterobacter vulneris	12.5	Damaged	*Cellulomonas massiliensis*	12.5	Undamaged
Sphingomonas insulae	12.5	Damaged	*Peptoniphilus obesi*	12.5	Undamaged
Spirosoma fluviale	12.5	Damaged	*Pseudonocardia atypica*	12.5	Undamaged
Bacteroides dorei	12.5	Damaged	*Pontibacter rhizosphera*	12.5	Damaged
Lactobacillus kitasatonis	12.5	Damaged	*Kocuria assamensis*	12.5	Undamaged
Spirilliplanes yamanashiensis	12.5	Undamaged	*Pedobacter kyungheensis*	12.5	Undamaged
Microbacterium saccharophilum	12.5	Damaged	*Piscicoccus nakaumiensis*	12.5	Undamaged
Lactobacillus delbrueckii	12.5	Damaged	*Leucobacter kyeonggiensis*	12.5	Damaged
Agromyces sp.ANK073	12.5	Damaged	*Gemella taiwanensis*	12.5	Undamaged
Xylophilus ampelinus	12.5	Damaged	*Hymenobacter ruber*	12.5	Damaged
Erwinia sp.JCM	12.5	Damaged	*Ralstonia suwonense*	12.5	Damaged
Kaistia soli	12.5	Damaged	*Paracoccus communis*	12.5	Undamaged
Planococcus donghaensis	12.5	Damaged	*Sphingomonas daechungensis*	12.5	Undamaged
Clostridium XlVa sp.RK1P	12.5	Undamaged	*Pedobacter pallidicorallinus*	12.5	Undamaged
Agaricicola sp.DMGB13	12.5	Undamaged	*Klebsiella quasipneumoniae*	12.5	Damaged
Deinococcus radiophilus	12.5	Damaged	*Pedobacter huanghensis*	12.5	Undamaged
Sphingomonas yunnanensis	12.5	Damaged	*Oceanicola sabulilitoris*	12.5	Undamaged
Rhizobacter sp.CB	12.5	Damaged	*Thermomonas carbonis*	12.5	Undamaged
Terrimonas sp.CB	12.5	Damaged	*Rhodopseudomonas gotjawalensis*	12.5	Damaged
Microvirga sp.CB	12.5	Damaged	*Veillonella seminalis*	12.5	Damaged
Ameyamaea sp.C37	12.5	Damaged	*Flavobacterium qiangtangense*	12.5	Damaged
Jiangella sp.1011TES3C79	12.5	Damaged	*Blastococcus dictyosporus*	12.5	Undamaged
Caulobacter mirabilis	12.5	Damaged	*Microbacterium halimionae*	12.5	Undamaged
Phycicoccus sp.URHC0019	12.5	Damaged	*Olsenella scatoligenes*	12.5	Undamaged
Afipia bacterium	12.5	Damaged	*Pontibacter humi*	12.5	Undamaged
Collinsella sp.GM6	12.5	Undamaged	*Clostridium XI sedimentorum*	12.5	Damaged
Clostridium XlVa sp.AT9	12.5	Damaged	*Sphingomonas gei*	12.5	Undamaged
Aminobacter aminovorans	12.5	Damaged	*Amnibacterium soli*	12.5	Damaged
Staphylococcus pasteuri	12.5	Undamaged	*Mesorhizobium jarvisii*	12.5	Damaged
Variovorax boronicumulans	12.5	Damaged	*Microbacterium proteolyticum*	12.5	Damaged
Rhodobacter sp.MST15SBBC	12.5	Damaged	*Serratia chamberiensis*	12.5	Damaged
Burkholderia sp.LMG	12.5	Damaged	*Paracoccus sanguinis*	12.5	Damaged
Planococcus sp.1304-W20	12.5	Damaged	*Collimonas alpina*	12.5	Damaged
Sanguibacter sp.1309-W7	12.5	Damaged	*Staphylococcus petrasii*	12.5	Undamaged
Methanobrevibacter smithii	12.5	Undamaged	*Paracoccus olei*	12.5	Damaged
Lactobacillus ruminis	12.5	Undamaged	*Intrasporangium nitratireducens*	12.5	Undamaged
Bacteroides uniformis	12.5	Damaged	*Dactylosporangium cerinum*	12.5	Undamaged
Parasutterella excrementihominis	12.5	Undamaged	*Novosphingobium gossypii*	12.5	Damaged
Actinoplanes consettensis	12.5	Damaged	*Diaphorobacter polyhydroxybutyrativorans*	12.5	Undamaged
Aquabacterium commune	12.5	Undamaged	*Rhizobium lentis*	12.5	Undamaged
Subtercola boreus	12.5	Damaged	*Tetrasphaera terrae*	12.5	Undamaged
Cellvibrio fulvus	12.5	Damaged	*Paracoccus angustae*	12.5	Damaged
Arthrobacter albus	12.5	Damaged	*Bifidobacterium ramosum*	12.5	Undamaged
Pseudonocardia spinosispora	12.5	Damaged	*Corynebacterium sp.16S*	12.5	Damaged
Haemophilus pittmaniae	12.5	Undamaged	*Bacillus subtilis*	12.5	Undamaged
Leifsonia pratensis	12.5	Undamaged	*Solibacillus silvestris*	12.5	Undamaged
Pedobacter cryoconitis	12.5	Undamaged	*Pseudomonas brenneri*	12.5	Undamaged
Arsenicicoccus bolidensis	12.5	Damaged	*Bacillus mycoides*	12.5	Both

species	% discovery across samples	Isolation surface	species	% discovery across samples	Isolation surface
Nocardioides aestuarii	12.5	Damaged	*Brevibacillus brevis*	12.5	Damaged
Granulicatella adiacens	12.5	Undamaged	Arthrobacter protophormiae	12.5	Damaged
Actinobacillus porcinus	12.5	Undamaged	Paenibacillus lactis	12.5	Damaged
Turicella otitidis	12.5	Undamaged	Isoptericola variabilis	12.5	Damaged
Agromyces ramosus	12.5	Undamaged	Sporosarcina saromensis	12.5	Both
Micrococcus lylae	12.5	Undamaged	Bacillus sporothermodurans	12.5	Damaged
Dermabacter hominis	12.5	Undamaged	Bacillus sp.BC11	12.5	Undamaged
Corynebacterium riegelii	12.5	Undamaged	Paenibacillus lautus	12.5	Damaged
Facklamia ignava	12.5	Damaged	Bacillus safensis	12.5	Both
Clostridium sensu stricto disporicum	12.5	Undamaged	Bacillus sp.PVS08	12.5	Undamaged
Rothia amarae	12.5	Undamaged	Paenibacillus pabuli	12.5	Damaged
Pseudomonas salomonii	12.5	Undamaged	Bacillus niacini	12.5	Damaged
Lactobacillus gastricus	12.5	Damaged	Lysinibacillus fusiformis	12.5	Damaged
Nesterenkonia lutea	12.5	Damaged	Paenibacillus sp.19783	12.5	Damaged
Bifidobacterium saeculare	12.5	Damaged	Bacillus coagulans	12.5	Damaged

	Raw reads	Merged paired end reads	Total contigs	Contigs with >120x coverage	Good's Coverage Estimator
BUP-1D	965050	343464	3961	359	0.88
BUP-1U	946256	371254	6672	281	0.86
BOL-1D	1348468	495878	11441	524	0.86
BOL-1U	1136930	407879	5111	342	0.86
LCA-2D	780062	288692	6864	377	0.86
LCA-2U	922652	362587	7044	461	0.86
PAG-1D	999314	196087	2661	273	0.88
PAG-1U	914296	360839	6110	380	0.86
Total	8013028	2826680	49864	2997	

Supplementary Table 2: Breakdown of the metagenomic sequencing data for each site and sampling coverage results.

Primer name	Primer sequence
16S-rRNA_F27	AGAGTTTGATCMGGC
16S-rRNA_R1525	AAGGAGGTGWTCCARCC
MS_BACT-16S_For	GGATTAGATACCCTGGTAGTCC
MS_BACT-16S_Rev	TCGTTGCGGGACTTAACCCAAC
PS_16S_F555	CTCCTACGGGAGGCAGCAGT

Supplementary Table 3: Primers used for PCR and sequencing of the 16S rRNA genes from bacterial studies. 16S-rRNA-F27 and 16S-rRNA-R1525 are universal primers commonly used for this purpose. MS_BACT-16S_For and MS_BACT-16S_Rev are primers designed by, and used with permission of Dr. Michael Shaw, University of Lincoln. All primers were produced by Sigma. PS_16S_F555 was designed by the author.

						Protimeter		pH		
		Relative Humidity (%)	Temperature (Celcius)	Light (lux)	UV (uW/lumen)	Damaged	Undamaged	Damaged	Undamaged	Aspect
	Relative Humidity (%)	x	-0.77 / p0.0001	-0.495 / p=.0365	0.5589 / p=.015	0 / p>0.05	0 / p>0.05	0 / p>0.05	0 / p>0.05	0 / p>0.05
	Temperature (Celcius)	-0.77 / p0.0001	x	0.6938 / p=0.001	-.686 / 9=0.001	0 / p>0.05	0 / p>0.05	0 / p>0.05	0 / p>0.05	0 / p>0.05
	Light (lux)	-0.495 / p=.0365	0.6938 / p=0.001	x	-0.736 / p=.0005	0 / p>0.05	0 / p>0.05	0 / p>0.05	0 / p>0.05	0 / p>0.05
	UV (uW/lumen)	0.5589 / p=.015	-.686 / 9=0.001	-0.736 / p=.0005	x	0 / p>0.05	0 / p>0.05	0 / p>0.05	0 / p>0.05	0 / p>0.05
Protimeter	Damaged	0 / p>0.05	0 / p>0.05	0 / p>0.05	0 / p>0.05	x	0 / p>0.05	0.7653 / p=0.0002	0 / p>0.05	0 / p>0.05
	Undamaged	0 / p>0.05	0 / p>0.05	0 / p>0.05	0 / p>0.05		x	0 / p>0.05	0 / p>0.05	0 / p>0.05
pH	Damaged	0 / p>0.05	0 / p>0.05	0 / p>0.05	0 / p>0.05	0.7653 / p=0.0002	0 / p>0.05	x	0 / p>0.05	0 / p>0.05
	Undamaged	0 / p>0.05	0 / p>0.05	0 / p>0.05	0 / p>0.05	0 / p>0.05	0 / p>0.05	0 / p>0.05	x	
	Aspect	0 / p>0.05	0 / p>0.05	0 / p>0.05	0 / p>0.05	0 / p>0.05	0 / p>0.05	0 / p>0.05	0 / p>0.05	x

Supplementary Table 4: Analysis of environtmental factors by Pearsons correlation co-efficient.

Supplementary Figure 1: Biofilm sampling record sheets used to record sample location, environmental conditions and other associated factors. One sheet was used for each pair of samples.

Biofilm sampling record sheet

Building name: St Bottophs Church	Building location: Lincoln
Name of recorder: Philip [illegible]	Date and time of sampling: 15/9/15 14:54
No. samples taken: 2 1D 1U	Sample code: BOL
Location within building: East End North facing corner	RH: 50.6 % Temp: 26.4 degC<br>Light: 5834 lux UV: 4 uW/lumen

Notes re. location i.e. gutters, water flow, vegetation proximity:

Gutter above in good condition

Sketch of area including damage, sample locations, measurements in mm:

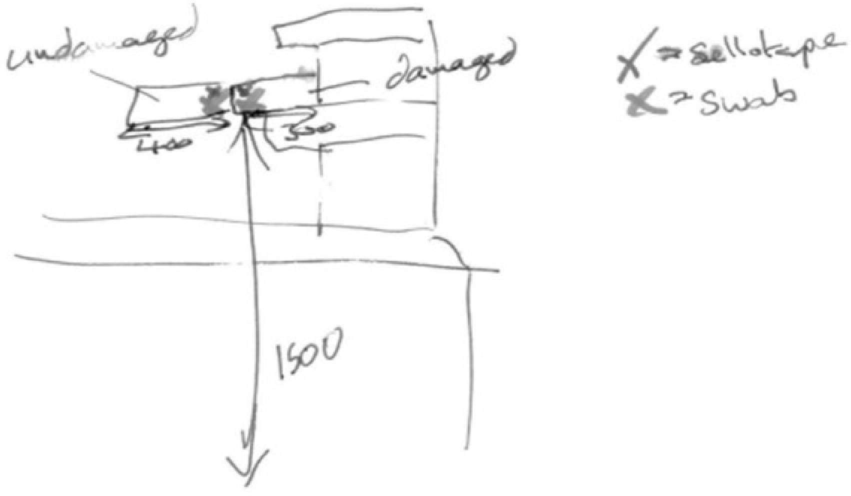

pH: U 6
D 5.5

Protimeter reading: U 10
D 16

Biofilm sampling record sheet

Building name: The Blessed Virgin St. Nicholas, St. Andrew & St Mary Church	Building location: Burton Pedwardine
Name of recorder: Philip Skipper	Date and time of sampling: 15/9/15 13:08
No. samples taken: 2 1 damaged 1 undamaged	Sample code: BuP
Location within building: WSW wall of NW chancel	RH: 72.8 % Temp: 17.1 degC<br>Light: 9324 lux UV: 1273 uW/lumen

Notes re. location i.e. gutters, water flow, vegetation proximity:

gutter above in good condition.

Sketch of area including damage, sample locations, measurements in mm:

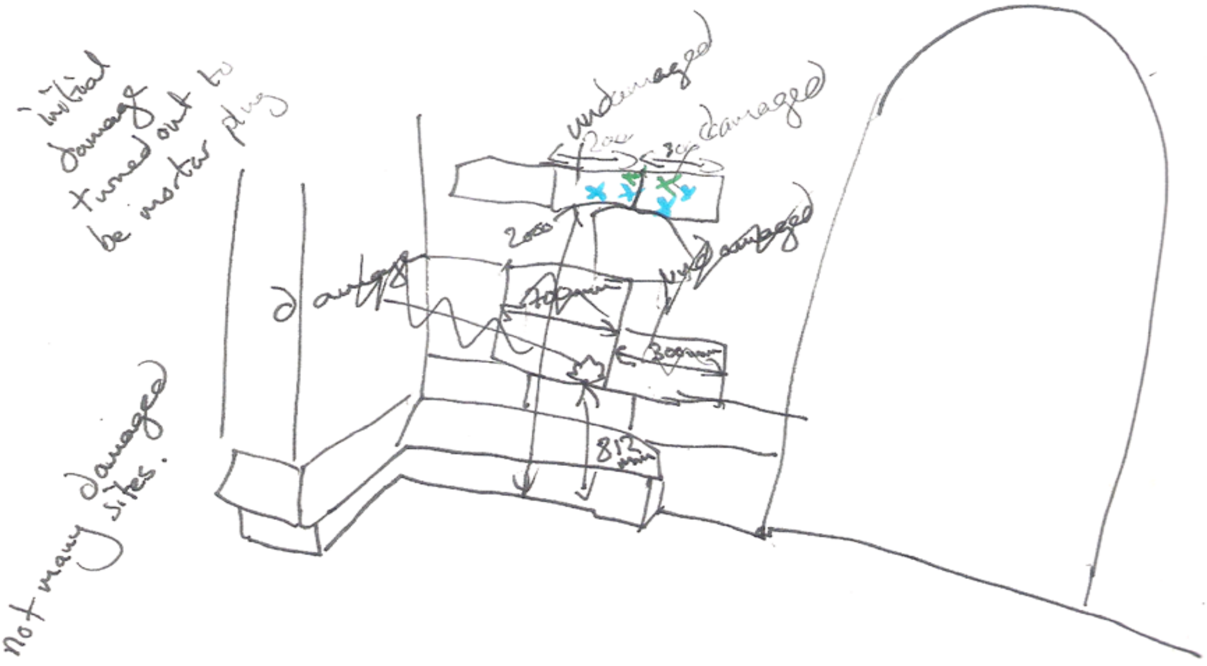

2.56km from A17 (1.6miles)

pH: V 6
D 5

Protimeter reading: U 12
D 24

Biofilm sampling record sheet

Building name: St Peter @ Gowts	Building location: Lincoln High Street
Name of recorder: Lynda Skipper	Date and time of sampling: 7/3/13 10.30
No. samples taken: 2 sellotape: 2 swab	Sample code: PAGU/D
Location within building: South facing buttress rear of side chapel	RH: 50 % Temp: [illegible] degC<br>Light: 4700 lux UV: 800 uW/lumen

Notes re. location i.e. gutters, water flow, vegetation proximity:
Vegetation none. No water draining down. Exposed site.

← 53 cm →

Sketch of area including damage, sample locations, measurements in mm:

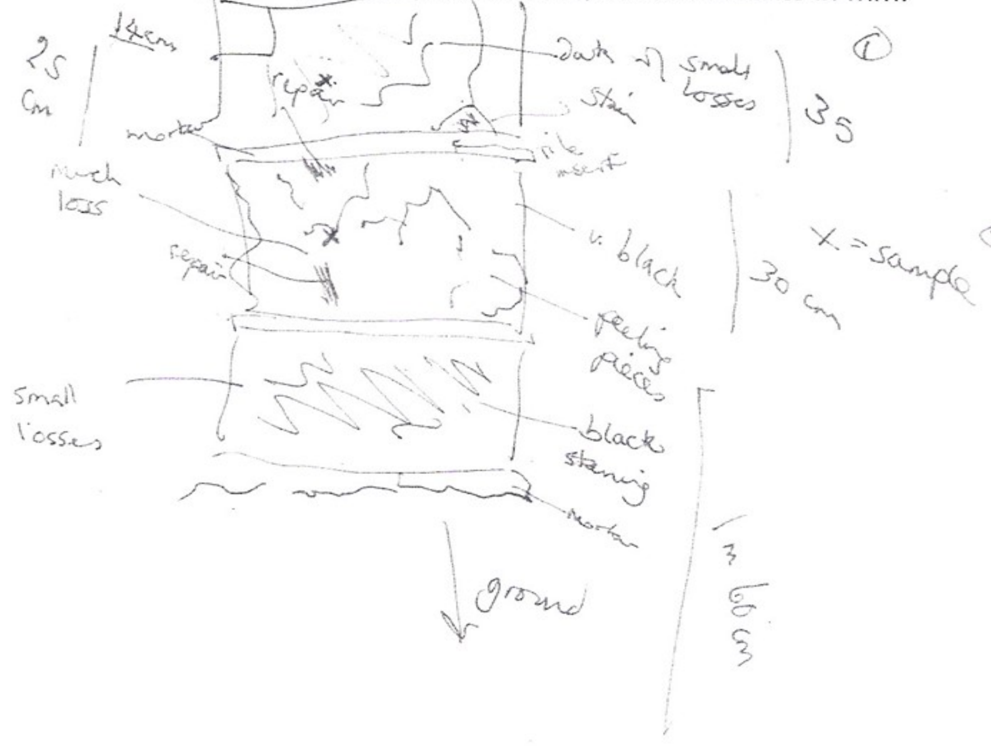

Most cement mortar

pH: ① 6 long range 5.5–6 short range
② 5 [illegible] e 4.5 short range

Protimeter reading: ① 17–18
② 18

Biofilm sampling record sheet

Building name: Cathedral	Building location: Lincoln
Name of recorder: Lynda Skipper	Date and time of sampling: 10.25 17/4/13
No. samples taken: 2	Sample code: LCAU/D
Location within building: East face of SW transept buttress Opp toilet block on older cath. wall	RH: 83.4% Temp: 12.2 degC<br>Light: 500 lux UV: 800 uW/lumen

Notes re. location i.e. gutters, water flow, vegetation proximity:

None

Sketch of area including damage, sample locations, measurements in mm:

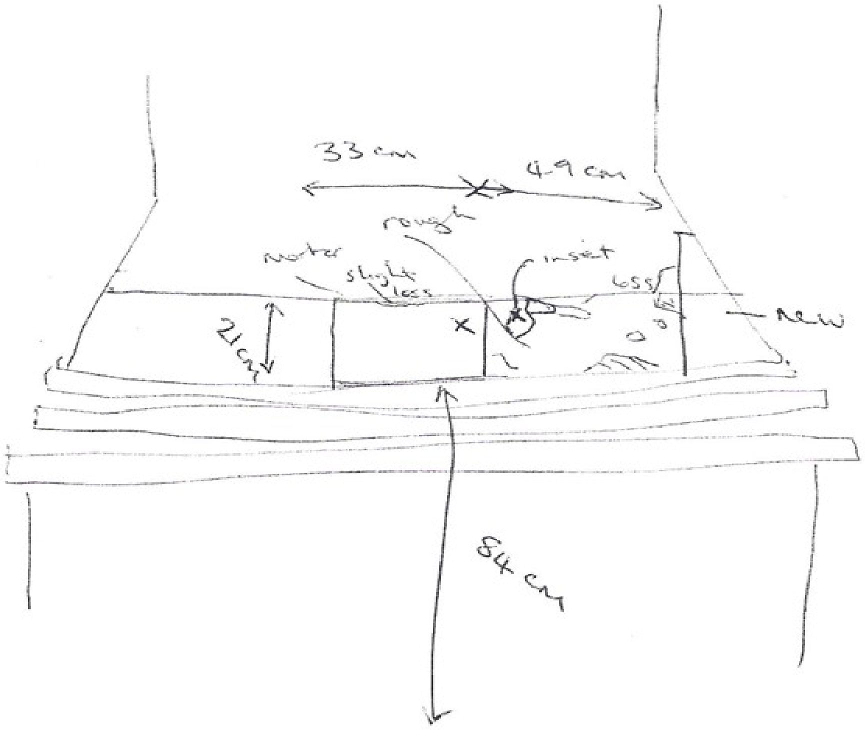

pH: 5.5 damaged<br>6 undamaged	Protimeter reading: 18 damaged<br>15 undamaged
